# The C-Type Lectin Receptor DC-SIGN Has an Anti-Inflammatory Role in Human M(IL-4) Macrophages in Response to *Mycobacterium tuberculosis*

**DOI:** 10.3389/fimmu.2018.01123

**Published:** 2018-06-12

**Authors:** Geanncarlo Lugo-Villarino, Anthony Troegeler, Luciana Balboa, Claire Lastrucci, Carine Duval, Ingrid Mercier, Alan Bénard, Florence Capilla, Talal Al Saati, Renaud Poincloux, Ivanela Kondova, Frank A. W. Verreck, Céline Cougoule, Isabelle Maridonneau-Parini, Maria del Carmen Sasiain, Olivier Neyrolles

**Affiliations:** ^1^Institut de Pharmacologie et de Biologie Structurale, IPBS, Université de Toulouse, CNRS, UPS, Toulouse, France; ^2^International Associated Laboratory (LIA) CNRS “IM–TB/HIV” (1167), Toulouse, France; ^3^International Associated Laboratory (LIA) CNRS “IM–TB/HIV” (1167), Buenos Aires, Argentina; ^4^IMEX-CONICET, Academia Nacional de Medicina, Buenos Aires, Argentina; ^5^Centre for Genomic Regulation, Barcelona, Spain; ^6^Department of Surgery, University, Hospital Erlangen, Friedrich-Alexander, University Erlangen-Nürnberg, Erlangen, Germany; ^7^INSERM/UPS/US006 CREFRE, CHU Purpan, Toulouse, France; ^8^Biomedical Primate Research Centre, Rijswijk, Netherlands

**Keywords:** macrophages, *Mycobacterium tuberculosis*, DC-SIGN, C-type lectin receptors, anti-inflammatory

## Abstract

DC-SIGN (CD209/CLEC4L) is a C-type lectin receptor (CLR) that serves as a reliable cell-surface marker of interleukin 4 (IL-4)-activated human macrophages [M(IL-4)], which historically represent the most studied subset within the M2 spectrum of macrophage activation. Although DC-SIGN plays important roles in *Mycobacterium tuberculosis* (Mtb) interactions with dendritic cells, its contribution to the Mtb–macrophage interaction remains poorly understood. Since high levels of IL-4 are correlated with tuberculosis (TB) susceptibility and progression, we investigated the role of DC-SIGN in M(IL-4) macrophages in the TB context. First, we demonstrate that DC-SIGN expression is present both in CD68^+^ macrophages found in tuberculous pulmonary lesions of non-human primates, and in the CD14^+^ cell population isolated from pleural effusions obtained from TB patients (TB-PE). Likewise, we show that DC-SIGN expression is accentuated in M(IL-4) macrophages derived from peripheral blood CD14^+^ monocytes isolated from TB patients, or in macrophages stimulated with acellular TB-PE, arguing for the pertinence of DC-SIGN-expressing macrophages in TB. Second, using a siRNA-mediated gene silencing approach, we performed a transcriptomic analysis of DC-SIGN-depleted M(IL-4) macrophages and revealed the upregulation of pro-inflammatory signals in response to challenge with Mtb, as compared to control cells. This pro-inflammatory gene signature was confirmed by RT-qPCR, cytokine/chemokine-based protein array, and ELISA analyses. We also found that inactivation of DC-SIGN renders M(IL-4) macrophages less permissive to Mtb intracellular growth compared to control cells, despite the equal level of bacteria uptake. Last, at the molecular level, we show that DC-SIGN interferes negatively with the pro-inflammatory response and control of Mtb intracellular growth mediated by another CLR, Dectin-1 (CLEC7A). Collectively, this study highlights a dual role for DC-SIGN as, on the one hand, being a host factor granting advantage for Mtb to parasitize macrophages and, on the other hand, representing a molecular switch to turn off the pro-inflammatory response in these cells to prevent potential immunopathology associated to TB.

## Introduction

According to the latest World Health Organization (WHO) report, tuberculosis (TB) is the largest killer among communicable diseases (WHO Annual report 2017). In 2016, there were an estimated 1.7 million deaths due to TB, making it the leading cause of death worldwide due to a single infectious agent, *Mycobacterium tuberculosis* (Mtb). In general, it is estimated that one quarter of the human population could be latently infected with Mtb (1). The bacillus may be active either after infection or through the reactivation of latent infection, which occurs in approximately 5% of infected people. During latency, for which there are no pathological or contagious conditions, Mtb is contained within elaborated aggregates of immune cells that are called granulomas, the hallmark of TB (2, 3). It is thought that a dedicated immune response is responsible for the formation and maintenance of granulomas, which will ultimately determine the outcome of the disease (2, 4). However, there is a strong need to better understand the factors that define an efficient immune response both during the early and late phases of Mtb infection in order to facilitate potential targets for preventive and therapeutic purposes.

Macrophages are considered key players during the early and late stages of Mtb infection (5). These sentinel cells are strategically located in secondary lymphoid organs and multiple mucosal sites, such as lung alveolar and interstitial space. At such, macrophages recognize and internalize Mtb and, consequently, modulate the inflammatory response to shape their microenvironment (e.g., granulomas) and the adaptive immune response against this pathogen. Interestingly, these cells display a high degree of tissue heterogeneity within the broad spectrum of pro- (M1) and anti-inflammatory (M2) programs of activation that manifest intracellular pathogen resistance and permissiveness, respectively (6). Macrophages may also serve as long-lived pathogen tissue reservoirs and contribute to TB pathogenesis (6–9). Remarkably, Mtb influences the differentiation, maturation, and activation of macrophages, resulting in the circumvention of the immune system and augmented persistence in the host (6–8, 10). This capacity of Mtb to modulate the host pro-inflammatory response and seize the anti-inflammatory mechanisms has generated a keen interest to investigate how this pathogen manipulates the process of macrophage activation.

The initial interaction with Mtb is thought to be crucial for macrophage activation and the eventual disease outcome. Pattern recognition receptors (PRRs) expressed in macrophages determine the binding, internalization, and fate of the bacillus’ intracellular lifestyle. Among the various PRR families that recognize Mtb, the C-type lectin receptors (CLR) are known to contribute to the control or persistence of this pathogen within macrophages (11–13). The CLR family includes collectins, selectins, endocytic and phagocytic receptors, and proteoglycans. CLRs are calcium-dependent glycan-binding proteins exhibiting similarities in the structures of the carbohydrate-recognition domain (CRD), which in turn recognize the carbohydrates expressed on the surface of Mtb including glycolipids [e.g., phosphatidyl-*myo*-inositol mannoside (PIM)], glycoglycans [e.g., lipoarabinomannan (LAM)], polysaccharides (e.g., α-glucan) and glycoproteins (e.g.,19 kDa antigen). In recent years, our understanding of the interactions of mycobacterial ligands with CLRs has advanced considerably, specifically in membrane-anchored phagocytic receptors such as the mannose receptor MRC1 (CD206/CLEC13D), Dectin-1 (CLEC7A) and Dectin-2 (CLEC6A), Mincle (CLEC4E), and Dendritic Cell-Specific Intercellular adhesion molecule-3-Grabbing Non-integrin (DC-SIGN/CD209/CLEC4L) (11–15). In general terms, the activation of these receptors by Mtb leads to downstream effects like endocytosis, oligomerization, intracellular trafficking, and signal transduction. Emerging evidence points out that signaling pathways triggered by these CLRs converge in a limited set of synergistic or antagonistic interactions with other PRRs and with each other, giving rise to a phenomenon known as signaling crosstalk. Interestingly, Mtb has evolved the capacity to subvert CLR signaling crosstalk to increase its survival and fitness within macrophages (5, 11, 15).

Studies by others and us identified and characterized DC-SIGN as a key receptor for Mtb in human dendritic cells and alveolar macrophages (14, 16–20). This CLR is a transmembrane receptor that possesses one single extracellular CRD (at the C-terminus) capable of recognizing mannose-containing molecules such as those present in mycobacterial Man-LAM (mannose-capped LAM), lipomannan (LM), arabinomannan, glycoproteins (e.g., 19, 38, and 45 kDa antigens), PIMs, and α-glucan, among others. Its functional cytoplasmic domain (at the N-terminus) contains different motifs that are crucial for endocytosis/phagocytosis, intracellular trafficking, and signal transduction (11). In the case of Mtb, it was shown that DC-SIGN is targeted by the mycobacterial ManLAM to induce the immunosupressive mediator interleukin-10 (IL-10) and counteract the toll-like receptor-4 (TLR-4)-dependent pro-inflammatory response (14). In this manner, the bacillus prevents the proper activation of dendritic cells given that IL-10 inhibits the expression of co-stimulatory molecules (e.g., CD86) and production of IL-12, which are essential for the activation of type-1 immunity best represented by T-helper 1 (Th1) cells. Of note, the hijacking of IL-10 production *via* DC-SIGN in dendritic cells seems to be a general evasion strategy by various pathogens like *Mycobacterium leprae, Helicobacter pylori*, and *Candida albicans*, among others (14).

Until recently, the expression of DC-SIGN was thought to be exclusive to human dendritic cells. In fact, this CLR is also expressed in human alveolar macrophages and some lymphocyte populations (19, 21). In particular, DC-SIGN is a reliable cell-surface marker in human macrophages activated with interleukin 4 (IL-4) [M(IL-4)], which is historically the most representative subset within the M2 spectrum of macrophage activation (22, 23). While the role of M(IL-4) macrophages has not been explored in in the TB context, high levels of type-2 inflammatory signals, such as IL-4, are correlated to TB susceptibility and progression (10). Reciprocally, the predominant type-2 inflammatory environment shifts toward type-1 immune signals [e.g., interferon-γ (IFNγ)] upon successful treatment of pulmonary TB. This is important because, in comparison to IFNγ, IL-4 renders macrophages less microbicidal against intracellular bacterial infections (6, 24). Furthermore, our group has also reported that DC-SIGN expression is induced specifically in alveolar macrophages of patients with active TB (19), suggesting a major role for this CLR in the interaction between macrophages and Mtb.

In this study, we provide for the first-time evidence supporting the anti-inflammatory role of DC-SIGN in the M(IL-4) macrophage response to Mtb. We report that DC-SIGN expression is accentuated in macrophages under different contexts using samples from TB patients, or in tuberculous pulmonary lesions of non-human primates (NHP), arguing for the pertinence of DC-SIGN-expressing macrophages in TB pathology. In the absence of this CLR, M(IL-4) macrophages displayed a pro-inflammatory signature upon challenge with Mtb, and acquired a better ability to control the intracellular growth of this pathogen. Finally, while there are no major changes in the production of IL-10, we demonstrate that DC-SIGN interferes negatively with the activation of M(IL-4) macrophages triggered by Dectin-1.

## Materials and Methods

### Ethics Statement for Non-Human Primate Samples

The NHP study protocol was done to comply with the EC Directive 86/609/EEC, approved by the local independent ethics committee prior to the start of the study, and executed under Dutch law on animal experiments (agreement number DEC#579). The endpoint for any particular animal was based either by signs of severe disease (humane endpoint criteria, referring to animal condition by adverse body weight development, respiratory capacity, and animal behavior) or by protocol, which limited the follow-up time to 1-year postinfection.

### Non-Human Primate Handling

The NHP samples were prepared from animals that were used for vaccine research and development purposes, as previously described (25). Briefly, healthy young adult female rhesus macaques (*Macaca mulatta*), all captive-bred for research purposes and of homogeneous breeding background, were challenged with 500 colony forming units (CFU) of Mtb strain Erdman K01 (prepared and provided under an agreement between WHO and CBER/FDA with assistance of Aeras), which was administered by intra-bronchial instillation under sedation. At endpoint, the animals were sedated, euthanized and submitted to macroscopic lung pathology scoring, as previously published (26).

### Histological Analyses on Non-Human Primate Samples

The gross pathological findings were assessed and described by an experienced veterinary pathologist while blinded for treatment as previously described (25). Representative lung biopsies were collected and fixed in 10% neutral buffered formalin and embedded in paraffin for long-term storage. Tissue sections were stained with hematoxylin and eosin (HE) for histomorphological analysis. Histopathological scoring of TB lesions in NHP was determined using a worksheet in which TB disease from lung biopsies was described (25, 26). Immunohistochemical staining was performed on paraffin-embedded tissue sections using the following monoclonal primary antibodies: CD68 (clone:KP1, Dako), DC-SIGN (clone 120612, R&D System), CD163 (clone 10D6, Leica/Novocastra), and MerTK (clone: Y323, Abcam). After incubation with primary antibodies, sections were stained with biotin-conjugated polyclonal anti-mouse or -rabbit immunoglobulin antibodies followed by the streptavidin-biotin-peroxidase complex (ABC) method (Vector Laboratories), and then were counterstained with hematoxylin. Slides were scanned with the Panoramic 250 Flash II (3DHISTECH). For confocal microscopy, samples were stained with primary antibodies as described above and followed by anti-mouse IgG isotype specific or anti-rabbit IgG antibodies labeled with Alexa488 and Alexa555 (Molecular Probes). Samples were mounted with Prolong^®^ Antifade reagent (Molecular Probes) and examined using a 40×/0.95N.A. objective of an Olympus FV1000 confocal microscope.

### Ethics Statement for Human Samples

Blood samples from healthy subjects (HS) or TB patients were provided by the Blood Transfusion Service, Hospital Fernandez, Buenos Aires (agreement number CEIANM-52-5-2012), or the Hospital F. J. Muñiz, Buenos Aires (protocol number: NIN-1671-12). Pleural effusions (PE) were obtained by therapeutic thoracentesis by physicians at the Hospital F. J. Muñiz (Buenos Aires). The research was carried out in accordance with the Declaration of Helsinki (2013) of the World Medical Association and was approved by the Ethics Committees of the Hospital F. J. Muñiz and the Academia Nacional de Medicina de Buenos Aires (protocol number: NIN-1671-12). Written informed consent was obtained before sample collection. The diagnosis of TB pleurisy was based on a positive Ziehl–Nielsen staining or Lowestein–Jensen culture from PE and/or histopathology of pleural biopsy and was further confirmed by an Mtb-induced IFN-γ response and an ADA-positive test (27). Mononuclear cells from peripheral blood (PB) and PE were isolated by Ficoll-Hypaque gradient centrifugation (Pharmacia, Uppsala, Sweden), as described previously (25, 28).

Likewise, monocytes from HS were isolated from buffy coat provided by Etablissement Français du Sang, Toulouse, under contract 21/PLER/TOU/IPBS01/2013-0042. According to articles L1243-4 and R1243-61 of the French Public Health Code, the contract was approved by the French Ministry of Science and Technology (agreement number AC 2009-921). Donors signed and provided written informed consents before sample collection.

### Preparation of Pool of Sera and Tuberculous PE

The cell-free supernatant from tuberculous PE and sera was transferred into new plastic tubes, further centrifuged at 12,000 *g* for 10 min and aliquots were stored at −80°C. Pools were prepared by mixing same amounts of eight individual PE or serum. The pools were de-complemented at 56°C for 30 min, and filtered by 0.22 µm in order to remove any remaining debris or residual bacteria.

### Preparation of Human Monocyte-Derived Macrophages From HS and TB Patients

Monocytes from HS or TB patients were isolated and differentiated into macrophages as previously described (25, 28). Briefly, purified CD14^+^ monocytes from HS were differentiated for 5–7 days in RPMI-1640 medium (GIBCO), 10% fetal bovine serum (FBS, Sigma-Aldrich), and human recombinant macrophage colony-stimulating factor (M-CSF, Peprotech) at 10 ng/mL. The cell medium was renewed every 3 or 4 days. Thereafter, macrophages were treated with IL-4 (Peprotech) at 20 ng/mL to induce the M(IL-4) program, 10% v/v of a pool of sera from HS or TB patients, or of acellular fraction of TB PE for 48 h. Untreated cells under differentiation with M-CSF only were considered as part of the M(M-CSF) program.

Alternatively, monocyte isolation and purification was done as previously published (29). Succinctly, following Ficoll gradient enrichment, monocytes were purified using positive selection with anti-CD14 microbeads and MACS separation columns (Miltenyi Biotec), according to manufacturer’s instructions. For macrophage differentiation, monocytes were allowed to adhere to glass coverslips (VWR international) in 6-well or 24-well plates (Thermo Scientific), at 1.5 × 10^6^ and 3 × 10^5^ cells/well, respectively, for 2 h at 37°C in warm RPMI-1640 medium (GIBCO). The medium was then supplemented to a final concentration of 10% FBS (Sigma-Aldrich) and human recombinant M-CSF (Peprotech) at 20 ng/ml, and the cells were allowed to differentiate for 5 days. The cell medium was renewed at day 3 or 4 of culture. At day 5, macrophages were activated for 48 h with 1 µg/ml of lipopolysaccharide (LPS, Invivogen) and 2.5 ng/ml of IFN-γ (Miltenyi Biotec) to induce the M(LPS + IFN-γ) program, and with 20 ng/ml of IL-4 (Miltenyi Biotec) for the M(IL-4) program.

### Flow Cytometry

Cells from TB patients and related controls from HS (2 × 10^5^ cells) were labeled as described above and acquired in a FACSAria II cytometer (BD Biosciences). The monocyte–macrophage population was gated according to its forward scatter (FSC) and size scatter (SSC) proprieties. The percentage of positive cells and the median fluorescence intensity (MFI) were analyzed using FCS Express V3 software (De Novo Software, Los Angeles, CA, USA). In the context of macrophage activation programs, the CLR expression was analyzed by flow cytometry using antibodies (general dilution of 1:400) against DC-SIGN and Dectin-1 from R&D Systems, and MRC1 from BD Pharmingen (San Diego, CA, USA). Concerning the cellular content in PE fluid from TB patients, DC-SIGN expression was also analyzed by flow cytometry in mononuclear cells, gating within the CD14^+^ population and using human anti-DC-SIGN (R&D Systems).

Alternatively, the antibody staining of macrophages was performed as previously described (29). Adherent cells were collected using the Cell Dissociation Buffer according to manufacturer’s instructions (Life Technologies), centrifuged for 5 min at 340 *g* at 4°C, and then stained in cold FACS buffer (PBS pH 7.2, 5% BSA) for 25 min with the indicated fluorophore-conjugated antibodies using a general dilution of 1:400. The antibodies were the following: CD16 (FCGR3A), PD-L1 (CD274), CD11b (MAC-1) and CD14 from Biolegend; CD163 (SCARI1), CD86 (B7-2), CD64 (FCGR1A), CD36 (SCARB3), CD11c (ITGAX), IL-7R (CD127), MRC1, and DC-SIGN from BD Biosciences; MerTK (Tyro12) from R&D system, and HLA-DR from Santa Cruz Biotechnology. In parallel, we also performed the staining with the corresponding isotype control antibody. Afterward, the cells were washed with cold FACS buffer, centrifuged for 5 min at 340 *g* at 4°C, and analyzed by flow cytometry using LSR-II flow cytometer (BD Biosciences). Data was then acquired and analyzed using the FlowJo 7.6.5 software.

### siRNA Silencing of DC-SIGN and Dectin-1

The siRNA gene silencing in human primary macrophages was performed using the forward transfection approach, as previously described (29). Briefly, macrophages differentiated at day 5 were transfected using the lipid-based HiPerfect system (Qiagen) and an ON-TARGETplus SMARTpool siRNA targeting DC-SIGN (siDC-SIGN) and Dectin-1 (siDectin-1), or a non-targeting siRNA (siControl) (Dharmacon), at a final concentration of siRNA at 200 nM. Of note, for the simultaneous inactivation of two genes, the working siRNA concentration combined for DC-SIGN and Dectin-1 did not exceed the final concentration of 200 nM, as previously published (29, 30). After 6 h, RPMI-1640 medium supplemented with M-CSF (10 ng/ml) was added to each well, and the cells were allowed to recuperate overnight. The following day, IL-4 (20 ng/ml) was added to the transfected macrophages in order to induce the M(IL-4) program along with the CLR expression for an additional 72 h (or as indicated). The inactivation for DC-SIGN and Dectin-1 was confirmed by flow cytometry in non-permeabilized cells at the indicated time points post-transfection. Cell viability was determined by using the Annexin-V-FITC kit designed for flow cytometry. Cell viability was defined as cells negative for either Annexin-V-FITC and/or propidium iodide. For the upregulation of co-stimulatory molecules, control and DC-SIGN-depleted M(IL-4) macrophages were stimulated with LPS (1 µg/ml) for 24 h, and the cell-surface marker expression was assessed by flow cytometry.

### Blocking and Stimulation of DC-SIGN and Dectin-1 Receptors

Human macrophages were differentiated at day 5, and activated with IL-4 (20 ng/ml) for additional 48 h. At day 7, the M(IL-4) macrophages were washed with warmed RPMI-1640 medium supplemented with M-CSF (10 ng/ml) and pre-incubated for 30 min with 10 µg/ml blocking antibodies specific to either Dectin-1 (MAB1859, R&D Systems) or DC-SIGN (ab13847, abcam), or both. As a control, M(IL-4) macrophages were incubated with an irrevelevant antibody (Ab-Control). For efficient stimulation of Dectin-1, M(IL-4) macrophages were treated with cytochalasin D (1 µg/ml, C8273, SIGMA) in combination with purified β-glucan from Saccharomyces cerevisiae (10 µg/ml, G5011, SIGMA), as previously described (31); for DC-SIGN, cells were stimulated with 10 µg/ml of ManLAM (kindly provided by Dr. Jerome Nigou, IPBS/CNRS); and for TLR-4, cells were stimulated with 1 µg/ml of LPS. After 24 h, the supernatants were collected and stored at −80°C until further use for ELISA analysis.

### Mtb Strain, Culture, and Preparation for Infection

All manipulation with Mtb (H37Rv strain) was performed in a dedicated BSL-3 laboratory. Mtb was cultured at 37°C in Middlebrook 7H9 medium (Difco) supplemented with 10% albumin-dextrose-catalase (Difco) and 0.05% Tween-80 (Sigma-Aldrich). For infection, exponentially growing Mtb was centrifuged (2,000 *g*) for 15 min, and resuspended in 1x phosphate buffered saline (PBS). Clumps were dissociated by passages through a 26-G needle, and then resuspended in RPMI-1640 containing 10% FBS. The mycobacterial concentration was determined by measuring optical density at 600 nm [OD_600_]. For binding experiments, the GFP-expressing Mtb (H37Rv) strain was generated and cultivated as previously published (20).

### RNA Extraction and Transcriptomic Analysis

Control and DC-SIGN-depleted M(IL-4) macrophages (approximately 1.5 million cells) were infected with Mtb at a multiplicity of infection (MOI) of 3 bacteria to 1 cell in RPMI-1640 with 10% FBS for 4 h. The cells were then washed twice with 1x PBS. At this point, the cells were either treated with TRIzol Reagent (Invitrogen) to harvest at 4 h postinfection (*p.i*.) and stored at −80°C, or cultured with RPMI-1640 with 10% FBS overnight. The procedure with the TRIzol reagent was repeated the following day to harvest at 18 h *p.i*. and stored at −80°C. Total RNA was extracted from the TRIzol samples using the RNeasy mini kit (Qiagen). The amount and purity of RNA (absorbance at 260/280 nm) was measured with the Nanodrop ND-1000 apparatus (Thermo Scientific). Complementary DNA was reverse transcribed from 1 µg total RNA with Moloney murine leukemia virus reverse transcriptase (Invitrogen) using random hexamer oligonucleotides for priming, according to the manufacturer’s protocol. The microarray analysis was done using the Agilent Human GE 4 × 44 v2 (single color), as previously described (32). Briefly, the hybridization was performed with 2 µg Cy3-cDNA and the hybridization kit (Roche NimbleGen). According to manufacturer’s protocol, the samples were incubated for 5 min at 65°C, and 5 min at 42°C before loading for 17 h at 42°C. After washing, the microarrays were scanned with MS200 microarray scanner (Roche NimbleGen). Using Feature Extraction software, the Agilent raw files were extracted and then processed through Bioconductor (version 3.1) in the R statistical environment (version 3.2.0). A careful assessment of the quality of the hybridization, evaluation of the sampling method and normalization of the expression values was done as previously published (32). We then obtained the differentially expressed genes (DEGs) between control and DC-SIGN-depleted M(IL-4) macrophages at each time point after Mtb infection based on false discovery rate (*t*-test, *P* < 0.1) and a threshold of twofold change in the comparison between the two conditions. The normalized values for the entire microarray analysis and the determined DEGs are provided in Tables S1–S3 in Supplementary Material. Finally, DEGs were analyzed with QIAGEN’s Ingenuity^®^ Pathway Analysis (IPA^®^, QIAGEN Redwood City, www.qiagen.com/ingenuity).

### qRT-PCR Analysis

The amplification of RNA was performed with an ABI Prism 7500 Sequence Detector (Applied Biosystems) using the PCR SYBR Green sequence detection system (Eurogentec, Seraing, Belgium). Primers are listed in Table S4 in Supplementary Material. Data were analyzed using the software supplied with the Sequence Detector (Applied Biosystems). The mRNA content was normalized to the metastatic lymph node protein 51 (MLN51) mRNA and quantified using the ΔΔCt method.

### Semi-Quantitative Cytokine/Chemokine Antibody Array Assay and Quantitative ELISA

Control and DC-SIGN-depleted M(IL-4) macrophages were infected with Mtb at MOI of 3. Supernatants were harvested at 4 and 18 h p.i., centrifuged (2,000 *g*) for 10 min, passed through filters (0.22 µm pores) and stored at −80°C. With the use of the Human Cytokine Antibody Array C Series 1000 (RayBiotech), we assessed the supernatant content in terms of cytokine and chemokines following the manufacturer’s instructions, as previously described (33). We used Amersham Hyperfilm ECL (GE Healthcare) to detect individual signals, and the GS-800 calibrated densitometer (Bio-Rad) to quantify these signals. As instructed, the positive control signal on each array was used to normalize the rest of the detected signals. In parallel, cytokine quantification was measured in cell supernatants by ELISA using kits from BD Bioscience (TNFα, IL-6, and IL-10), according to manufacturer’s instructions.

### Binding and Phagocytosis of Mtb

As previously described (19), control and DC-SIGN-depleted M(IL-4) macrophages were infected with the GFP-expressing Mtb strain, at a MOI of 5 for 4 h, at either 4°C or 37°C, in order to assess binding and phagocytosis, respectively. Cells were then washed with 1× PBS (without calcium and magnesium), collected using the Cell Dissociation Buffer, centrifuged for 5 min at 340 *g* at 4°C, fixed with 4% PFA for 2 h, washed with 1× PBS and resuspended in FACS buffer. Cells were then analyzed by flow cytometry.

### Measurement of Mtb Intracellular Growth

Macrophages were washed with PBS and then infected with Mtb at a MOI of 0.2 bacteria/cell in RPMI-1640 with 10% FBS for 4 h. Cells were then washed twice with 1x PBS before addition of RPMI-1640/10% FBS. At the indicated time points, the cells were lysed in 0.01% Triton X-100 (Sigma-Aldrich), and serial dilutions of the lysates were plated onto 7H11-Oleic Albumin Dextrose Catalase (Difco) agar medium for CFU scoring.

### Statistical Analyses

Two-tailed Wilcoxon (matched-paired test) was applied to compare the M(IL-4) macrophage population under two conditions (siControl versus siDC-SIGN). Two-tailed unpaired *t*-test (parametric) was applied on data sets with a normal distribution, whereas one-tailed unpaired Mann–Whitney (nonparametric) was done for data not showing a normal distribution and where the outcome was already expected, as indicated for each figure legend. *P* < 0.05 was considered as the level of statistical significance.

## Results

### DC-SIGN-Expressing Macrophages Are Present in Pulmonary Lesions of NHP Infected With Mtb

**Figure 1:**
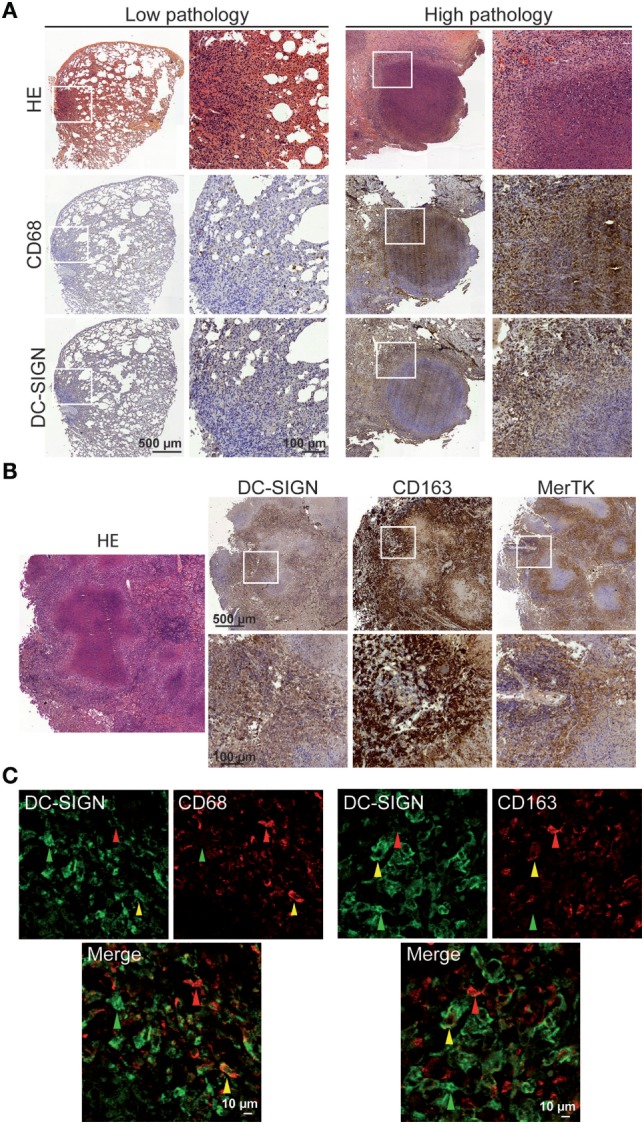
DC-SIGN-expressing macrophages are present in pulmonary lesions of non-human primates (NHP) infected with Mtb. **(A)** Representative immunohistochemical images illustrating the distribution of CD68 (middle row) and DC-SIGN (bottom row), among areas where leukocyte infiltration (top row) is detected by hematoxylin and eosin (HE), in pulmonary tissue and granulomas of NHP with very mild (left columns) and severe (right columns) pathology. **(B)** Representative immunohistochemical images illustrating the distribution of DC-SIGN, CD163, and MerTK in areas where leukocyte infiltration is detected by HE, such as in granulomas of NHP with severe pathology. **(C)** *Left panel*: immunostaining of DC-SIGN (green: Alexa-488) and CD68 (red: Alexa-555) in lung tissue of NHP with severe pathology; *right panel*: immunostaining of DC-SIGN (green: Alexa-488) and CD163 (red: Alexa-555) in lung tissue of NHP with severe pathology. Green arrow points out a cell positive for DC-SIGN only; red arrow a cell positive for CD68 or CD163 only; and yellow arrow for a cell positive for both DC-SIGN and CD68/CD163. Scale bar = 10 µm.

Tuberculous granulomas have organized microenvironments that presumably balance the antimicrobial functions to control bacteria growth and anti-inflammatory properties to limit pathology in the lung (34). Recently, others and we have detected the high abundance of an M2-like macrophage population in tuberculous granulomas in the context of pulmonary lesions of NHPs with severe TB (25, 35). Here, we further investigated whether DC-SIGN-expressing macrophages are present in these samples derived from Mtb-infected rhesus macaques, which displayed different levels of lung pathology. In samples from NHPs exhibiting a low pathological score in lungs, immunohistochemical analyses revealed the low presence of CD68^+^ macrophages and DC-SIGN-expressing cells in areas where leukocyte infiltrate was also detected by HE staining (Figure 1A). By contrast, in samples from animals characterized by a high lung pathological score, we observed not only the characteristic necrotic lung granuloma and CD68^+^ macrophages abundantly infiltrating the interstitial lung tissue, but also the increased number of DC-SIGN-expressing cells in these areas (Figure 1A). To determine whether DC-SIGN is expressed by M2 macrophages in the context of tuberculous granulomas, we also performed an additional staining for CD163 and MerTK, two strong markers of M2 macrophages. As shown for Figure 1B, DC-SIGN-expressing cells localized at the granuloma periphery and alveoli, where CD163^+^ and MerTK^+^ macrophages are also found. Importantly, co-localization analysis revealed the presence of cells positive for DC-SIGN within CD68^+^ (41 ± 20%, *n* = 893 counted cells) and CD163^+^ (27 ± 3%, *n* = 754 counted cells) macrophage populations (Figure 1C). These analyses demonstrate that the environment generated during pulmonary TB is associated with enhanced DC-SIGN expression in CD68^+^ and CD163^+^ macrophages, which become accentuated in NHPs with severe TB as previously described (25).

### Human Tuberculosis-Associated Microenvironment Induces DC-SIGN Expression in Macrophages

**Figure 2:**
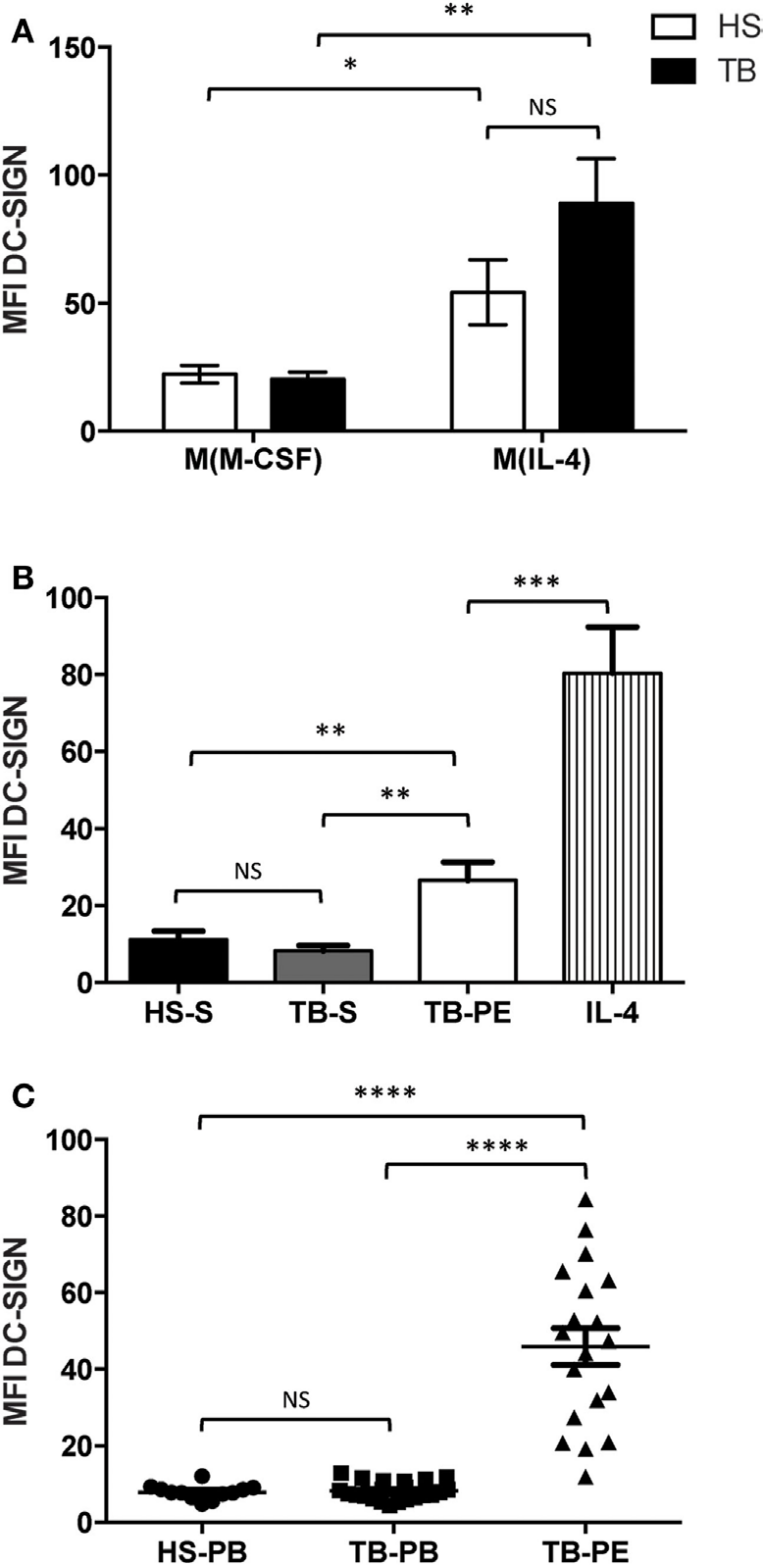
Human tuberculosis-associated microenvironment induces DC-SIGN expression in macrophages. **(A)** DC-SIGN expression is induced in M(IL-4) macrophages from TB patients. Freshly isolated monocytes from healthy subjects (HS, white) and TB patients (TB, black) were differentiated into macrophages using M-CSF. At day 5, the cells were activated with IL-4 (20 ng/ml) for 48 h to induce the M(IL-4) macrophage program. Otherwise, macrophages were kept under M-CSF to fully establish the M(M-CSF) program. The cells were harvested and the DC-SIGN expression was analyzed by flow cytometry. Vertical bar graphs depicting the median fluorescent intensity (MFI) of DC-SIGN expression in the different cell populations. Results are expressed as mean ± SD (*n* = 10 donors). **(B)** DC-SIGN expression is induced by pleural fluid from TB patients. Freshly isolated monocytes from HS were differentiated into macrophages using M-CSF. At day 5, the cells were activated for 48 h with sera from HS (HS-S, black) and TB patients (TB-S, gray), acellular pleural fluid from TB patients (TB-PE, white), and IL-4 (20 ng/ml, vertical stripes). The cells were harvested and the DC-SIGN expression was analyzed by flow cytometry. Vertical bar graphs depicting the MFI of DC-SIGN expression in the different cell populations. Results are expressed as mean ± SD (*n* = 13 donors). **(C)** DC-SIGN-expressing macrophages are present in the pleural cavity of TB patients. Mononuclear cells were isolated either from peripheral blood from HS (HS-PB) and TB patients (TB-PB), or from the pleural effusions from TB patients (TB-PE), and the expression of DC-SIGN was analyzed on the CD14^+^ population by flow cytometry. Results are expressed as vertical scatter plots showing the MFI of DC-SIGN for each population; each individual symbol represents a single donor. Two-tailed *t*-test (unpaired/parametric): *P < 0.05, **P < 0.01, ***P < 0.001, ****P < 0.0001, NS = not significant.

Our group previously reported that, in lungs, DC-SIGN expression is induced specifically in alveolar macrophages of patients with active TB (19). Using samples obtained from patients with active TB, we expanded our assessment of DC-SIGN expression in the context of macrophage activation. First, we isolated and differentiated monocytes from HS or TB patients into macrophages. Macrophages were activated toward the M(IL-4) program and the expression of DC-SIGN was measured by flow cytometry analysis. Our results revealed that, unlike unactivated macrophages [M(M-CSF)], DC-SIGN expression was accentuated in M(IL-4) macrophages and with a higher tendency (albeit not significant) in TB patients (Figure 2A). Second, we induced the activation of macrophages (derived from HS monocytes) with cell-free supernatants from sera or PE from TB patients (TB-S and TB-PE, respectively). We noticed that while sera from either HS or TB patients failed to induce DC-SIGN expression, TB-PE significantly increased the cell-surface level for this CLR (Figure 2B). Last, we measured DC-SIGN expression directly in the CD14^+^ cell population present in the circulation or PE of TB patients. We failed to observe the presence of DC-SIGN-expressing CD14^+^ blood monocytes in either HS or TB patients. Strikingly, however, we detected DC-SIGN highly expressed in all CD14^+^ monocyte/macrophages collected in TB-PE (Figure 2C). Altogether, these results demonstrate that TB-associated environments are capable to induce DC-SIGN expression in the context of human macrophage activation.

### Depletion of DC-SIGN Expression Does Not Affect Cell Viability nor the Establishment of the M(IL-4) Macrophage Program

In the context of the most commonly studied *in vitro* programs of human macrophage activation [i.e., M(LPS + IFNγ) and M(IL-4)], DC-SIGN is a reliable cell-surface marker for M(IL-4) macrophages (22, 23). To confirm this, we differentiated freshly isolated CD14^+^ monocytes from healthy donors into macrophages. We activated macrophages toward M(IL-4) and M(LPS + IFNγ), and then assessed the cell-surface marker profile of these cell populations by flow cytometry. While we noticed that expression of DC-SIGN and other markers varies dramatically between donors (in terms of MFI), we still detected a significantly higher cell-surface expression of DC-SIGN in M(IL-4) in comparison to M(LPS + IFNγ) macrophages (Figure S1A in Supplementary Material). Its expression was accompanied by that of Dectin-1 and MRC1, a *bona fide* M(IL-4) macrophage marker (22). We also observed a higher expression (albeit not significant) of CD11b and CD36 in M(IL-4) compared to M(LPS + IFNγ) cells (Figure S1A in Supplementary Material). By contrast, M(LPS + IFNγ) were distinguished by significant elevated levels of CD64, CD86, and PD-L1 (Figure S1A in Supplementary Material). These results confirm that DC-SIGN is exclusively expressed in M(IL-4) macrophages, thus making it an ideal cell model to study the role of this CLR in human macrophages.

With the aim to examine the role of DC-SIGN in the activation of the M(IL-4) program, we made use of a lipid-based siRNA-mediated gene silencing protocol we recently developed (29, 30). Macrophages were transfected with siRNA targeting this CLR (siDC-SIGN) or with non-targeting scrambled siRNA control (siControl), and these cells were then stimulated with IL-4 to activate the M(IL-4) program. As expected, while the MFI of DC-SIGN expression augmented under 72 h treatment with IL-4 in control cells, the induction of DC-SIGN was minimal in cells transfected with siRNA targeting this CLR (Figure S1B in Supplementary Material). In addition, cell viability was not affected after depletion of DC-SIGN for 72 h post-transfection (Figure S1B in Supplementary Material). Similar results in terms of DC-SIGN depletion and cell viability were obtained at 96, 120, and 144 h post-transfection (data not shown). Importantly, we noticed that DC-SIGN-depleted macrophages differentiate normally toward the M(IL-4) program (Figure S2A in Supplementary Material), and up-regulate co-stimulatory receptors when challenged with LPS (Figure S2B in Supplementary Material), as compared to control cells. Therefore, the inactivation of DC-SIGN in M(IL-4) macrophages appears to have no major consequences at steady state conditions or in response to LPS challenge.

### The Pro-Inflammatory Response of M(IL-4) Macrophages Against Mtb Is Regulated by DC-SIGN

**Figure 3:**
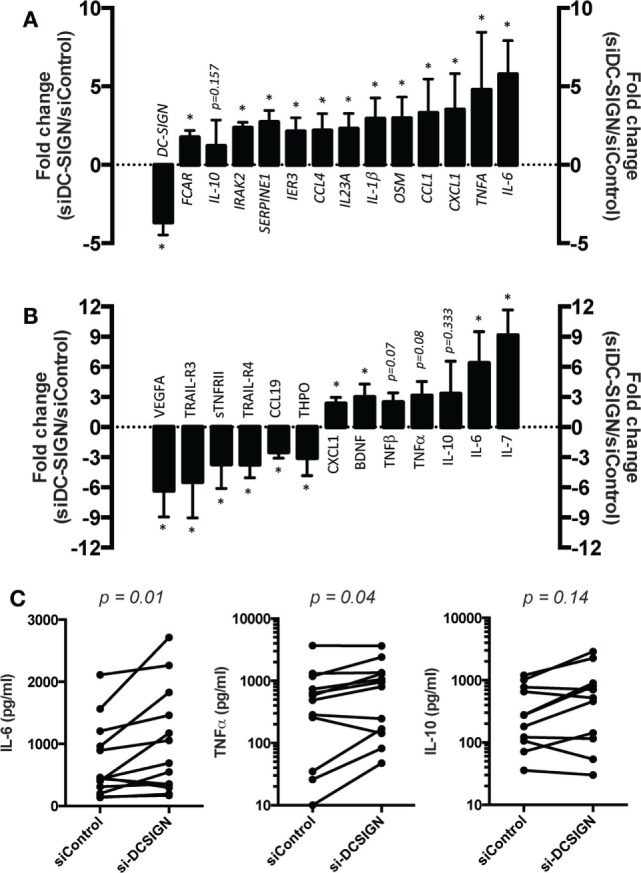
DC-SIGN regulates the pro-inflammatory response by M(IL-4) macrophages against Mtb. Human monocytes were differentiated into macrophages using M-CSF. At day 5, macrophages were transfected with siRNA targeting DC-SIGN (siDC-SIGN) or a non-targeting siRNA (siControl). The following day, the cells were activated with IL-4 (20 ng/ml) for 48 h to induce M(IL-4) program. The cells were then infected with Mtb at multiplicity of infection (MOI) of 3 bacteria to 1 cell. At 18 h *p.i*., the cells were harvested and their supernatant collected. Assessment of **(A)** gene mRNA expression by qRT-PCR analysis, and **(B)** cytokine and chemokine content by semi-quantitative antibody array. Vertical bar graphs illustrate the fold change of mRNA/protein levels in siDC-SIGN over siControl macrophages; “0” was set arbitrarily to represent no change. Results are expressed as mean ± SD (*n* = 4 donors). One-tailed Mann–Whitney (unpaired/nonparametric): *P < 0.05. **(C)** Assessment of cytokines by ELISA analysis. Results are expressed as before-and-after plot for the indicated genes (*n* = 11 donors). Each circle within the plots represents a single donor. Two-tailed Wilcoxon (matched-paired/nonparametric): *P* < 0.05 was considered as the level of statistical significance.

To explore the role of DC-SIGN in the M(IL-4) macrophage response to Mtb, we performed a genome-wide transcriptome analysis of cells expressing DC-SIGN (siControl) or not (siDC-SIGN) challenged with Mtb at different time points. In general, DC-SIGN-depleted M(IL-4) macrophages displayed a broad DEG profile when compared to control cells. At 4 h postinfection (*p.i*.), we observed that the majority of genes that are upregulated in DC-SIGN-depleted M(IL-4) macrophages are of pro-inflammatory nature, such as interferon alpha inducible protein (*IFI27*), *IL-6*, Oncostatin M (*OSM*), *CXCL1, IL-17RB*, and *IL-1β*, among others (Tables S1 and S2 in Supplementary Material). By contrast, we observed very few genes downregulated in DC-SIGN-depleted M(IL-4) macrophages, including *FCER1A* (*FcERI*), *CD1B, CCL17* (*TARC*), and DC-SIGN, among others (Tables S1 and S2 in Supplementary Material). At 18 h *p.i*., the pro-inflammatory tendency is further accentuated in the DEG that becomes upregulated in DC-SIGN-depleted M(IL-4) macrophages (Figure S3A and Tables S1 and S3 in Supplementary Material). For example, Ingenuity pathway analysis indicated that the DEG in these cells formed a network centered on NF-κB, a master transcription factor that regulates the pro-inflammatory response against infection (Figure S3A and Table S1 in Supplementary Material). Similar to 4 h *p.i*., there were few genes that were downregulated in in DC-SIGN-depleted M(IL-4) macrophages when compared to control cells at 18 h *p.i*., including DC-SIGN (Figure S3A and Tables S1 and S3 in Supplementary Material). Importantly, we confirmed the higher expression of the pro-inflammatory genes, and the downregulation of DC-SIGN, at 18 h *p.i*. by qPCR analysis (Figure 3A). Of note, the mRNA expression of *IL-10* was not affected significantly in the absence of DC-SIGN (Figure 3A).

At the protein level, we performed a dedicated antibody-based membrane array to assess the cytokine and chemokine content in the culture medium collected from DC-SIGN-depleted and control M(IL-4) macrophages at 18 h *p.i*. with Mtb. Again, we observed the shift toward a pro-inflammatory profile in DC-SIGN-depleted M(IL-4) macrophages as they secreted more IL-6, TNF, and CXCL1, among other cytokines and chemokines, compared to the supernatant content from siControl cells (Figure 3B). By contrast, the secretion of anti-inflammatory factors such as vascular endothelial growth factor A (VEGFA), thrombopoietin (THPO), TNF receptor superfamily member 10C (TNFRSF10C/TRAIL-R3) and 10D (TNFRSF10D/TRAIL-R4), among others, was downregulated in the absence of DC-SIGN (Figure 3B). Finally, we validated by ELISA analysis the high level of both IL-6 and TNF in the supernatant content of DC-SIGN-depleted M(IL-4) macrophages in comparison to that of control cells (Figure 3C). Similar to RNA levels, we observed no differences in the secretion of IL-10 in the absence of DC-SIGN, as measured both by the antibody-based membrane arrays and ELISA (Figures 3B,C).

Collectively, these results suggest that the expression of DC-SIGN restrain the pro-inflammatory capacity of M(IL-4) macrophages in response to Mtb.

### Inactivation of DC-SIGN Expression in M(IL-4) Macrophages Reduces the Intracellular Mtb Burden

**Figure 4:**
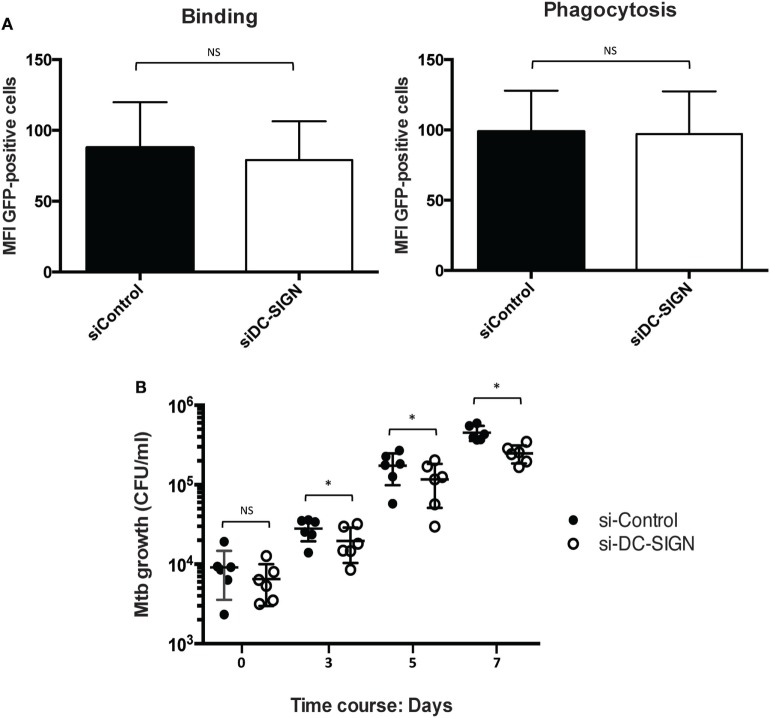
Different roles for DC-SIGN in the M(IL-4) macrophage–Mtb interaction. Human monocytes were differentiated into macrophages using M-CSF. At day 5, macrophages were transfected with siRNA targeting DC-SIGN (siDC-SIGN, white) or a non-targeting siRNA (siControl, black). The following day, the cells were activated for 48 h with IL-4 (20 ng/ml) to induce M(IL-4) program. **(A)** DC-SIGN expression in M(IL-4) macrophages is redundant for the binding and internalization of Mtb. Control and DC-SIGN-depleted macrophages were tested for the capacity to bind (at 4°C, left) or phagocytose (at 37°C, right) the Mtb strain expressing GFP during 4 h of challenge. Bar graphs illustrate the median fluorescent intensity (MFI) of cells positive for GFP, as measured by flow cytometry analysis. Results are expressed as mean ± SD (*n* = 4 donors). NS, not significant. **(B)** DC-SIGN influences the capacity of M(IL-4) macrophages to control the Mtb intracellular burden. The cells were infected with Mtb (MOI of 0.2 bacteria to 1 cell) and the intracellular growth of the bacteria was followed at 4 h (day 0), 72 h (day 3), 120 h (day 5), and 168 h (day 7), as measured by colony forming unit (CFU) assays. Results are expressed as vertical scatter plots showing the CFU scoring per ml; each circle represents a single donor. Two-tailed Wilcoxon (matched-paired/nonparametric): *P < 0.05; NS, not significant.

While macrophages from the M2 spectrum of activation are associated with intracellular pathogen permissivity (6), it was previously shown that activation of human macrophages with IL-4 enhances the microbicidal capacity against Mtb in a dose-dependent manner (24). To assess the role of DC-SIGN in the control Mtb intracellular growth, we first conducted flow cytometry-based experiments using a fluorescent Mtb strain (H37Rv-eGFP) to determine the capacity of M(IL-4) macrophages to bind and internalize the bacillus when DC-SIGN is inactivated. To our surprise, we noticed that both parameters were equally performed by control and DC-SIGN-depleted M(IL-4) macrophages (Figure 4A). Next, we carried out the CFU assays. At 4 h *p.i*., we confirmed that DC-SIGN-depleted M(IL-4) macrophages displayed normal levels of bacteria internalization compared to control cells (Figure 4B). However, when DC-SIGN is depleted, Mtb intracellular growth is better restricted at day 3, 5, and 7 *p.i*. compared to control M(IL-4) macrophages (Figure 4B). These results indicate that the expression of DC-SIGN renders M(IL-4) more permissive to intracellular growth by Mtb.

### DC-SIGN Interferes Negatively With the Activation of M(IL-4) Macrophages Triggered by Dectin-1 in Response to Mtb

**Figure 5:**
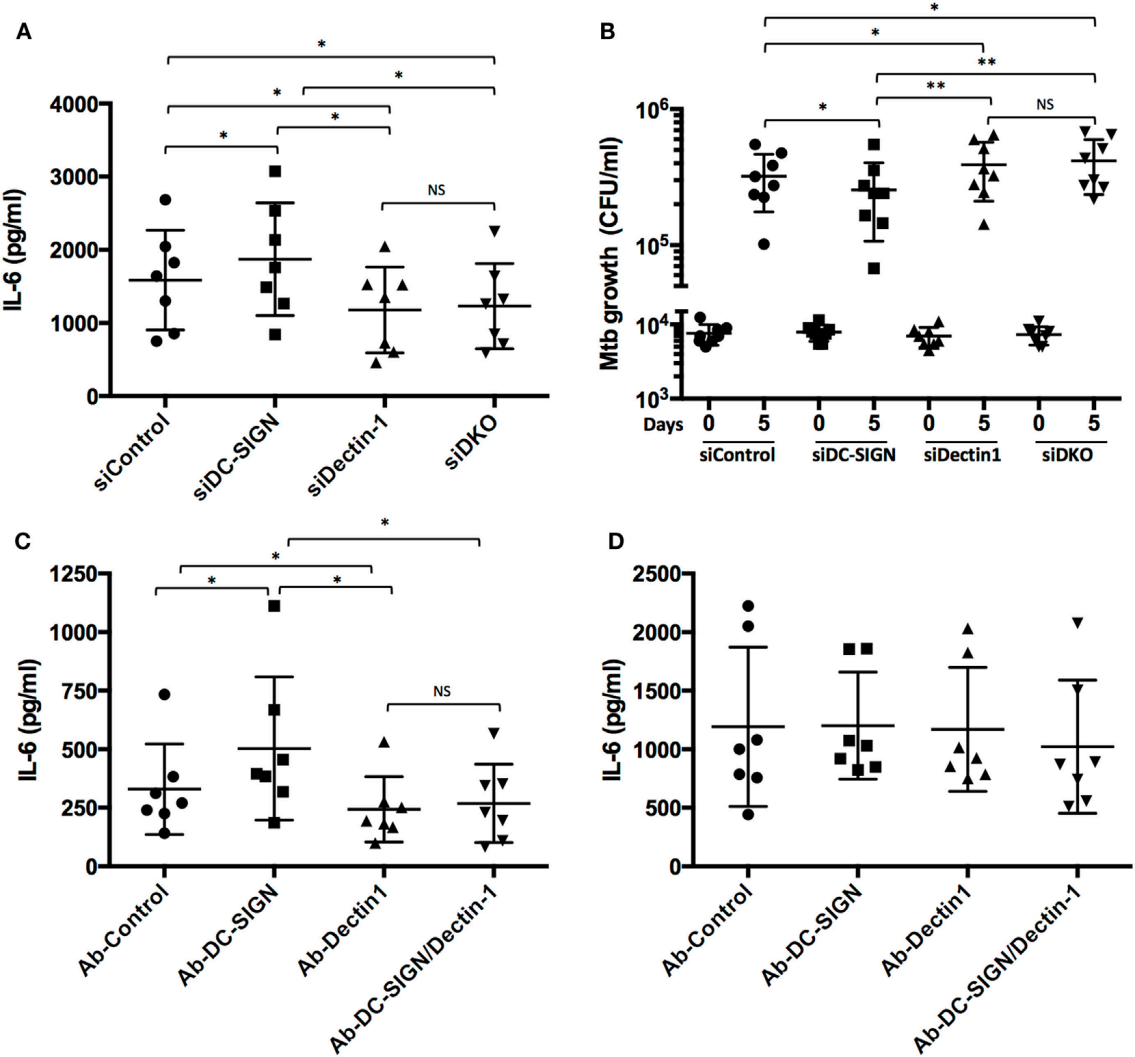
DC-SIGN interferes negatively with the activation of M(IL-4) macrophages triggered by Dectin-1. Human monocytes were differentiated into macrophages using M-CSF. At day 5, the macrophages were transfected with siRNA targeting DC-SIGN (siDC-SIGN, squares) and Dectin-1 (siDectin-1, upward triangle), both siRNAs (siDKO, downward triangle), or a non-targeting siRNA (siControl, circles). The following day, the cells were activated with IL-4 (20 ng/ml) for 48 h to induce M(IL-4) program and the C-type lectin receptor expression. **(A)** Assessment of IL-6 secretion by ELISA analysis. The cells were infected with Mtb at MOI of 3 bacteria to 1 cell. At 18 h *p.i*., the supernatant from these cells was collected. Results are expressed as vertical scatter plots, and as mean ± SD (*n* = 7 donors). **(B)** Assessment of Mtb intracellular growth by colony forming unit (CFU) assay. The cells were infected with Mtb (MOI of 0.2 bacteria to 1 cell), and the intracellular growth of the bacteria was followed at 4 h (day 0, left) or 120 h (day 5, right). Results are expressed as vertical scatter plots, and as mean ± SD (*n* = 8 donors). Two-tailed Wilcoxon (matched-paired/nonparametric): *P < 0.05, **P < 0.01; NS, not significant. **(C,D)** Upon differentiation until day 5, macrophages were then activated with IL-4 for 48 h. Prior to stimulation, M(IL-4) macrophages were pre-treated for 30 min with blocking antibodies for either DC-SIGN or Dectin-1, or both. An irrelevant antibody was used as a control. M(IL-4) macrophages were then treated with either **(C)** cytochalasin D (1 μg/ml), β-glucan (10 µg/ml) and ManLAM (10 µg/ml), or **(D)** LPS (1 µg/ml) and ManLAM (10 µg/ml). After 24 h, the supernatants were collected and the production of IL-6 was measured by for ELISA analysis.

Dectin-1 is an important CLR expressed in human dendritic cells that activates a pro-inflammatory response distinguished by IL-6, TNF, IL-23, and IL-1β, against Mtb (36). Using our siRNA-mediated gene silencing method (29), we inactivated the expression of Dectin-1 in M(IL-4) macrophages and confirmed its pro-inflammatory role in the response to challenge with Mtb, as reflected by the decrease of IL-6 secretion (as an example of the pro-inflammatory response) at 18 *p.i*. (Figure 5A; Figure S4 in Supplementary Material). Importantly, simultaneous inactivation of Dectin-1 and DC-SIGN resulted in a similar IL-6 secretion as Dectin-1 inactivation alone, indicating that the high levels observed for this cytokine in DC-SIGN-depleted M(IL-4) macrophages are dependent on the Dectin-1 signaling pathway (Figure 5A; Figure S4 in Supplementary Material). By contrast, the production of IL-10 was not affected by the inactivation of either DC-SIGN or Dectin-1 (Figure S5A in Supplementary Material). In terms of bacterial burden, we found that Dectin-1 depletion rendered the M(IL-4) macrophages more susceptible to Mtb growth (Figure 5B; Figure S4 in Supplementary Material). Moreover, simultaneous inactivation of both CLRs fully reversed the lower CFU counts observed when DC-SIGN is inactivated alone, suggesting that Dectin-1 depletion is dominant over that of DC-SIGN (Figure 5B; Figure S4 in Supplementary Material).

To better understand the potential crosstalk between these receptors in the generation of a pro-inflammatory response (e.g., IL-6 production), we employed the use of the ligands β-glucan and ManLAM to stimulate specifically Dectin-1 and DC-SIGN, respectively. As a control for macrophage activation, we used LPS as the ligand for TLR-4. As illustrated in the Figures 5C,D, we noticed that the stimulation of M(IL-4) macrophages with β-glucan and ManLAM induces the production of IL-6, albeit to a lower level than that obtained with LPS and ManLAM. Interestingly, while the use of a blocking antibody targeting DC-SIGN resulted in the upregulation of IL-6 after stimulation with β-glucan and ManLAM (Figure 5C), it failed to do so upon stimulation with LPS and ManLAM (Figure 5D). As expected, the use of a blocking antibody targeting the Dectin-1 receptor diminished the production of IL-6 induced by β-glucan and ManLAM (Figure 5C), but it had no effect on the production driven by the stimulation with LPS and ManLAM (Figure 5D). Likewise, simultaneous blocking of DC-SIGN and Dectin-1 resulted in a diminished production of IL-6 upon stimulation only with by β-glucan and ManLAM (Figure 5C), and not with LPS and ManLAM (Figure 5D). Of note, the crosstalk between DC-SIGN and Dectin-1 is specific for IL-6 because the production of the anti-inflammatory IL-10 was not affected (Figures S5B,C in Supplementary Material).

Altogether, these findings support a role of DC-SIGN in interfering negatively with the pro-inflammatory response triggered by Dectin-1 during Mtb infection of M(IL-4) macrophages.

## Discussion

This study highlights a dual role for DC-SIGN as, on the one hand, being a host factor granting advantage for Mtb to parasitize macrophages and, on the other hand, representing a molecular switch to turn off the pro-inflammatory response in these cells to potentially prevent potential immunopathology associated to TB. Notwithstanding, there are some limitations to this study that should be considered in the interpretation of results and derived conclusions. First, while our siRNA-mediated gene silencing protocol is effective in primary macrophages, without altering cell viability or biological functions (29), the expression of DC-SIGN is not totally abrogated. Prior to infection with Mtb, macrophages transfected with the siRNA targeting DC-SIGN still continue to express this CLR (albeit at minimal levels) upon activation with IL-4, ranging between 5 and 25% compared to control cells. Second, working with human primary macrophages introduces a high degree of biological variance compared to experiments done with human cell lines (e.g., THP-1) or bone marrow derived macrophages from congenic mice. Third, among membrane-anchored CLRs, there is a well-known functional redundancy that might cooperate in a coordinated immune response in favor or against Mtb infection (11, 37). Last, the context for which DC-SIGN is activated should be taken into account, as this CLR can mediate both immunosuppression and immunity (38). Indeed, there are multiple studies supporting the immunosuppressive role of DC-SIGN in macrophages during inflammatory contexts, including in autoimmunity and, just recently, in allotransplantation acceptance (38–40). Likewise, DC-SIGN is well known to play a pro-inflammatory role by enhancing the antigen-presentation process in human dendritic cells and inducing a strong activation of CD4^+^ and CD8^+^ T cells (41, 42). In the case of M(IL-4) macrophages, our findings on DC-SIGN should be taken within the immunosuppression context given that the nature of these cells is that of wound healers and tissue remodelers (22). In spite of these limitations, our study dealing with primary human cells in a TB context provides relevant data improving our knowledge on DC-SIGN.

We first described the presence of DC-SIGN-expressing macrophages in the pleural cavity of patients and in the lung tissue of NHP with active TB. The identification of a humanCD14^+^ macrophage population displaying high cell-surface levels of DC-SIGN, MRC1, and CD163, is supported by our previous finding describing the presence of the CD16^hi^CD163^hi^MerTK^hi^ immunosuppressive macrophages in the pleural cavity of patients with active TB (25), and by our first report on DC-SIGN-expressing alveolar macrophages isolated from the bronchoalveolar lavage in patients with pulmonary TB (19). This suggests that a TB-associated environment favors the presence of macrophages with an M2-marker signature, including DC-SIGN expression. Interestingly, IL-4 is one of the most detected cytokines in TB PE, and thus making probably the responsible signal to induce DC-SIGN expression in cells at this site (43). IL-13 is another cytokine that is present in the pleural cavity of active TB patients (44), and that is probably responsible for the mediating DC-SIGN expression in macrophages since its signaling pathway is considered to be equivalent to that of IL-4 (22). At the pulmonary tissue level, while we detected DC-SIGN-expressing cells with a morphology typical of dendritic cells and lymphocytes (likely B cells), there were also numerous DC-SIGN-expressing cells which also co-express the macrophage marker CD68 and CD163. This is supported by previous reports describing the presence of M2-like cells in the context of tuberculous granulomas structures (25, 34, 35). Beyond the TB context, CD68^+^ macrophages expressing DC-SIGN were preferentially detected in granuloma lesions in lepromatous leprosy (45), as well as in granuloma-like structures in pathological conditions of dermatological diseases, like granuloma annulare and necrobiosis lipoidica (46). Altogether, these findings confirmed the presence of DC-SIGN-expressing macrophages in different contexts of active TB, which may not be able to mount an appropriate type-1 immune response against Mtb infection, and thus may likely contribute to the pathogenesis of this disease. While limited, these findings provide an important association between the abundance of DC-SIGN expressing macrophages and active TB, and they highlight the need to establish whether these cells actually play a pathophysiological role in this disease.

We demonstrated that DC-SIGN regulates the pro-inflammatory response of M(IL-4) macrophages during Mtb infection. Indeed, this inflammatory profile closely resembles the common response of macrophages to bacterial infections involving the upregulation of genes typical within the spectrum of M1 macrophages (6). If excessive or prolonged, the M1 macrophage response could then be deleterious for the host in terms of tissue damage or organ failure, as demonstrated during *E. coli* infection in baboon experimental peritonitis (47). In addition, a recent study in human microglia supports the immunosuppressive role for DC-SIGN in macrophages against inflammatory insults (40). Garcia-Vallejo et al. elegantly demonstrated that this CLR interacts with fucosylated glycans on myelin oligodendrocyte glycoprotein resulting in the synergistic upregulation of the TLR4-dependent production of the anti-inflammatory cytokine IL-10 in these macrophages. However, to our surprise, the IL-10 production appears not to be responsible for the dysregulation of pro-inflammatory signals in M(IL-4) macrophages. In human dendritic cells, the activation of DC-SIGN with mycobacterial ManLAM (or agonist antibodies) leads to the synergetic increase of IL-10 if it coincides with TLR4 stimulation with bacterial LPS (17, 48). Based on this well-established crosstalk, we expected the levels of IL-10 to be significantly lower in DC-SIGN-depleted M(IL-4) macrophages compared to control cells, and thus explaining the tilting toward a pro-inflammatory nature. It is likely that human M(IL-4) macrophages differ from dendritic cells (and other cells) in this respect. This is supported by our previous study showing that DC-SIGN does not potentiate IL-10 secretion in LPS-stimulated alveolar macrophages from TB patients, which highly expressed this CLR along with TLR4 (19). Moreover, another key study using humanized DC-SIGN mice demonstrated the anti-inflammatory role of this CLR in macrophages conferring protection against autoimmunity in intravenous immunoglobulin therapy, which is dependent on IL-4 and IL-33 but not IL-10 (39). It is worth mentioning that the physiological role of TLR4 remains to be proven in the context of TB. While Mtb-derived compounds can activate TLR4 *in vitro* (49, 50), it does not appear to affect the *in vivo* immune response against Mtb, as demonstrated in the mouse model (51, 52). Beyond IL-10, we showed a downregulation of protein levels involved in angiogenesis and vascularization (e.g., VEGFA), thrombopoiesis (e.g., THPO), and anti-apoptosis factors (e.g., TRAIL-R3, TRAIL-R4), in the absence of DC-SIGN during Mtb infection. These results infer a decrease of homeostatic functions such as tissue repair and remodeling, which are hallmark functional properties of M(IL-4) macrophages (22, 53, 54), and they support an immunosuppressive role for DC-SIGN in M(IL-4) macrophages that seems independent on the potentiation of IL-10 production, which may represent yet another way for Mtb to control NF-κB-driven pro-inflammatory signals.

We determined that DC-SIGN expression in M(IL-4) macrophages affects their capacity to control the Mtb burden. To our knowledge, this is the first time that deficiency of DC-SIGN in human cells is shown to directly improve the control of bacterial load in the context of infection. This improved control of Mtb burden is neither due to defects in the differentiation or activation of M(IL-4) macrophages, nor due to deficient bacterial recognition or intake by these cells, in the absence of DC-SIGN. In terms of binding, we were greatly surprised that DC-SIGN-depleted M(IL-4) macrophages were able to bind and phagocytose Mtb at the same level as control cells. In the context of DC-SIGN-expressing alveolar macrophages from TB patients, and of the human monocytic cell line expressing DC-SIGN (THP-1:DC-SIGN), we convincingly demonstrated in the past that this CLR contributed majorly to the binding and infection of these cells by Mtb (19). However, one postulate governing M(IL-4) macrophages is the acquisition of an entirely different phagocytic receptor repertoire compared to other macrophages and cell types (22). In addition to DC-SIGN, these cells are characterized by MRC1, MSR1 (macrophage scavenger receptor 1), Dectin-1, DCIR (CLEC4A), DCL-1 (CLEC13A), MGL (CLEC10A), CD36, MS4A4A (CD20-L1), and CD23 (CLEC4J), among others. Thus, it is most likely that the absence of DC-SIGN is compensated by the high abundance of these receptors in M(IL-4) macrophages unlike other cells. Recently, we described that human DC-SIGN-depleted M(IL-4) macrophages became resistant to *Leishmania infantum* infection (30). This improved control of parasite infection was dependent on the high production of IL-1β in macrophages lacking DC-SIGN. In fact, we demonstrated that DC-SIGN downregulated the mRNA expression of *LTA4H* (leukotriene A4 hydrolase), whose enzymatic activity is critical for LTB4 (leukotriene B4) synthesis, and consequently, for the caspase-1-dependent production of IL-1β. In the TB context, this is not the case for DC-SIGN. While we confirmed a significant augmentation of the *IL-1β* mRNA, we did not observe a change in the levels of IL-1β and LTB4 proteins, nor in the *LTA4H* mRNA expression, in DC-SIGN-depleted M(IL-4) macrophages at 18 h *p.i*. (data not shown). Furthermore, there were no changes in mRNA expression of antimicrobial peptides (based on the transcriptomic data), the production of reactive oxygen species, nor in the autophagy flux, between DC-SIGN-depleted and control M(IL-4) macrophages at 18 h *p.i*. (data not shown). Therefore, we inferred there might be alternative microbicidal mechanisms affected by DC-SIGN expression in M(IL-4) macrophages to control the bacilli intracellular burden.

Finally, we shed light on the capacity of DC-SIGN to regulate the pro-inflammatory response and control of bacterial burden driven by Dectin-1. Both CLRs are expressed favorably in M(IL-4) macrophages. Our results demonstrate that Dectin-1 plays a pro-inflammatory and microbicidal role against Mtb. This is in agreement with the fact that Dectin-1 is an ITAM (immunoreceptor tyrosine-based activation motif) immunoreceptor with the capacity to engage a pro-inflammatory response upon engagement with its ligand, including through the activation NFκβ-dependent transcription profile (13). In the context of TB, Dectin-1-depleted mice exhibit reduction of bacterial burden in the lungs, but there are no differences in lung pathology score, cytokine levels, or survival (55). However, in human dendritic cells, Dectin-1 is clearly partially responsible for the pro-inflammatory response against Mtb infection, specifically in the induction of the Th1/Th17 immune response (36, 56). In the case of DC-SIGN, our results propose a role for this CLR to interfere negatively in the pro-inflammatory response to Mtb that is dependent on Dectin-1. The signaling crosstalk between Dectin-1 and DC-SIGN has been reported in the recent past (30, 36, 57). While this crosstalk can be synergistic such as in the case of prostaglandin 2 (PGE_2_) production in human dendritic cells, where these receptors are shown to even bind together (57), it can also be the opposite. In Mtb and *L. infantum* infection, DC-SIGN interferes negatively with the production of pro-inflammatory signals triggered by Dectin-1 in human dendritic cells and M(IL-4) macrophages, respectively (30, 36). In this study, while we cannot exclude contribution of other PRRs (e.g., TLR-2), we propose that the pro-inflammatory signature and improved control of Mtb burden in the absence of DC-SIGN must be mainly contributed by Dectin-1. Whether this signaling crosstalk can be translated to other human macrophage subsets expressing both CLRs remains to be confirmed.

In conclusion, most of what we know about DC-SIGN in the field of host–pathogen interaction mainly derives from the work done in human dendritic cells. While both dendritic cells and macrophages perform similar functions, these cells are thought to have different roles in the context of TB. On the one hand, dendritic cells are known as the professional antigen-presenting cell because they possess a great capacity to pick up and process antigens, migrate to secondary lymphoid organs, and present antigenic information to tailor an adaptive immune response against Mtb. On the other hand, macrophages are considered to be the premier effector because they are the first leukocyte to encounter Mtb in the alveolar space and activate the innate immune response to contain and eliminate this pathogen at the site of infection. Our present study brings into focus the anti-inflammatory role of DC-SIGN in M(IL-4) macrophages. Since type-2 inflammatory signals (e.g., IL-4, IL-13) are correlated with TB susceptibility and progression, we believe that DC-SIGN expression in these macrophages is of pertinence and consequence to TB pathogenesis. Indeed, these findings support the notion that Mtb hijacks the immunosuppressive aspects of DC-SIGN to invade and persist in M(IL-4) macrophages and, at the same time, modulate the local inflammatory response by these cells in its favor. Future studies shall focus on the identification of endogenous ligands targeting DC-SIGN to trigger the wound healing and tissue remodeling activity of M(IL-4) macrophages for the benefit of the patient in lung inflammatory disease. Furthermore, we believe these results can be extrapolated from the context of TB into parasitology, as M(IL-4) macrophages are considered as essential effector cells in parasite eradication (22). All things considered, beyond the role of DC-SIGN in macrophages, this study also points toward the need to investigate the pathophysiological impact of IL4 and other type-2 immune signals in the TB context, which remains unknown.

## Ethics Statement

Ethics Statement for Non-Human Primate Samples: In the Netherlands, the NHP study protocol was done to comply with the EC Directive 86/609/EEC, approved by the local independent ethics committee prior to the start of the study, and executed under Dutch law on animal experiments (agreement number DEC#579). The endpoint for any particular animal was based either by signs of severe disease (human endpoint criteria, referring to animal condition by adverse body weight development, respiratory capacity and animal behavior) or by protocol, which limited the follow-up time to 1-year postinfection. Ethic Statement for Human Samples: In Argentina, blood samples from HS or TB patients were provided by the Blood Transfusion Service, Hospital Fernandez, Buenos Aires (agreement number CEIANM-52-5-2012), or the Hospital F. J. Muñiz, Buenos Aires (protocol number: NIN-1671-12). PE were obtained by therapeutic thoracentesis by physicians at the Hospital F. J. Muñiz (Buenos Aires). The research was carried out in accordance with the Declaration of Helsinki (2013) of the World Medical Association, and was approved by the Ethics Committees of the Hospital F. J. Muñiz and the Academia Nacional de Medicina de Buenos Aires (protocol number: NIN-1671-12). Written informed consent was obtained before sample collection. The diagnosis of TB pleurisy was based on a positive Ziehl–Nielsen staining or Lowestein–Jensen culture from PE and/or histopathology of pleural biopsy, and was further confirmed by an Mtb-induced IFN-γ response and an ADA-positive test (27). Mononuclear cells from PB and PE were isolated by Ficoll-Hypaque gradient centrifugation (Pharmacia, Uppsala, Sweden), as described previously (25, 28). In France, monocytes from HS were isolated from buffy coat provided by Etablissement Français du Sang, Toulouse, under contract 21/PLER/TOU/IPBS01/2013-0042. According to articles L1243-4 and R1243-61 of the French Public Health Code, the contract was approved by the French Ministry of Science and Technology (agreement number AC 2009-921). Donors signed and provided written informed consents before sample collection.

## Author Contributions

Conceptualization and methodology: GL-V, AT, LB, CL, CC, and ON. Software: IM. Investigation: GL-V, AT, LB, CL, CD, IM, AB, FC, TS, RP, IK, and CC. Resources: FV, IM-P, MS, and ON. Writing: GL-V and ON. Visualization: TS, CC, and RP. Supervision: GL-V and ON. Corresponding author: GL-V.

## Conflict of Interest Statement

The authors declare that the research was conducted in the absence of any commercial or financial relationships that could be construed as a potential conflict of interest.
