# Supplementary figures and images for: The C-Type Lectin Receptor DC-SIGN Has an Anti-Inflammatory Role in Human M(IL-4) Macrophages in Response to Mycobacterium tuberculosis
Source: Front Immunol. 2018 Jun 12;9:1123. doi: 10.3389/fimmu.2018.01123 (PMC6006465; doi:10.3389/fimmu.2018.01123)

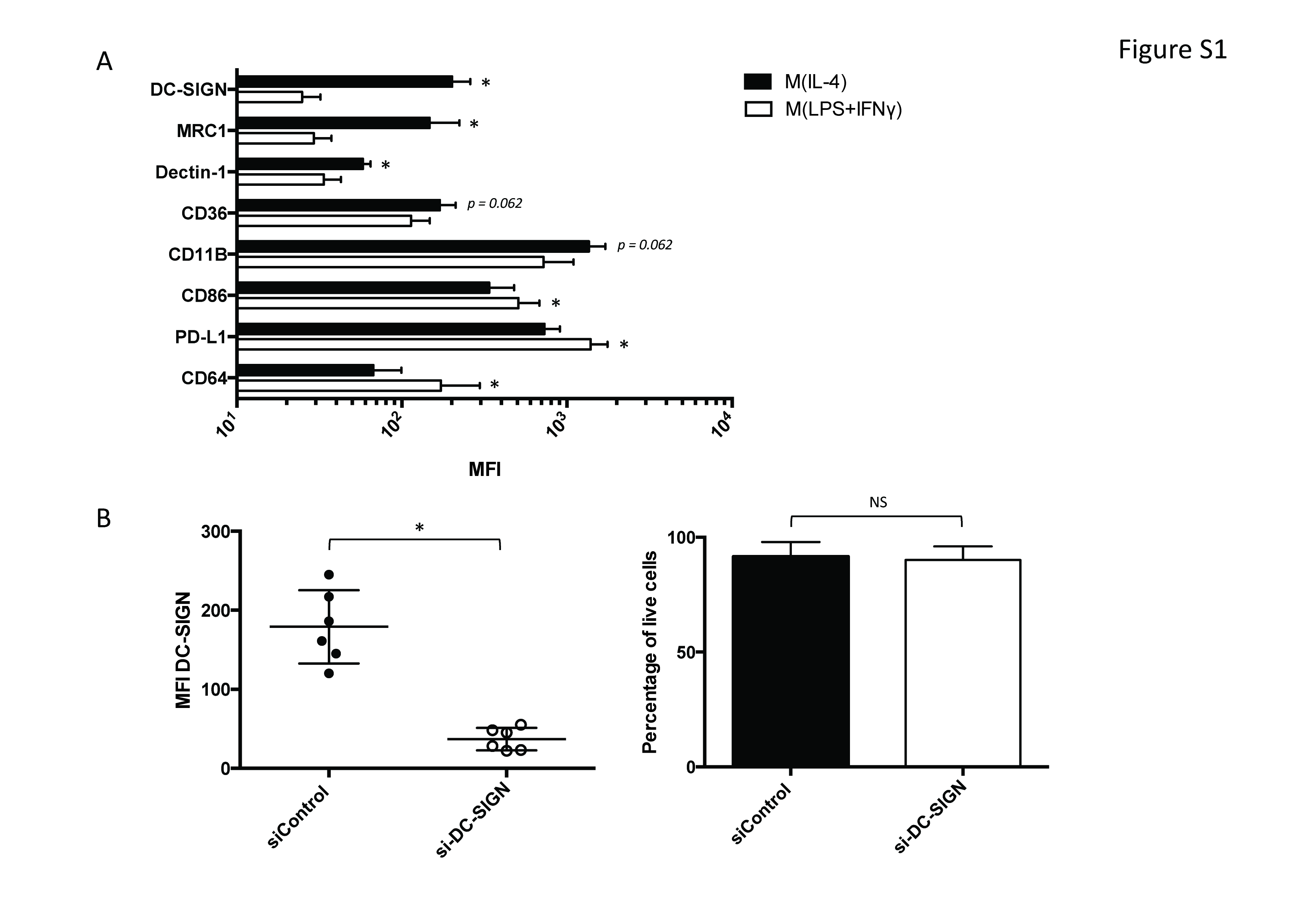

Supplement: Supplementary file 1 [file Image_1.tif]

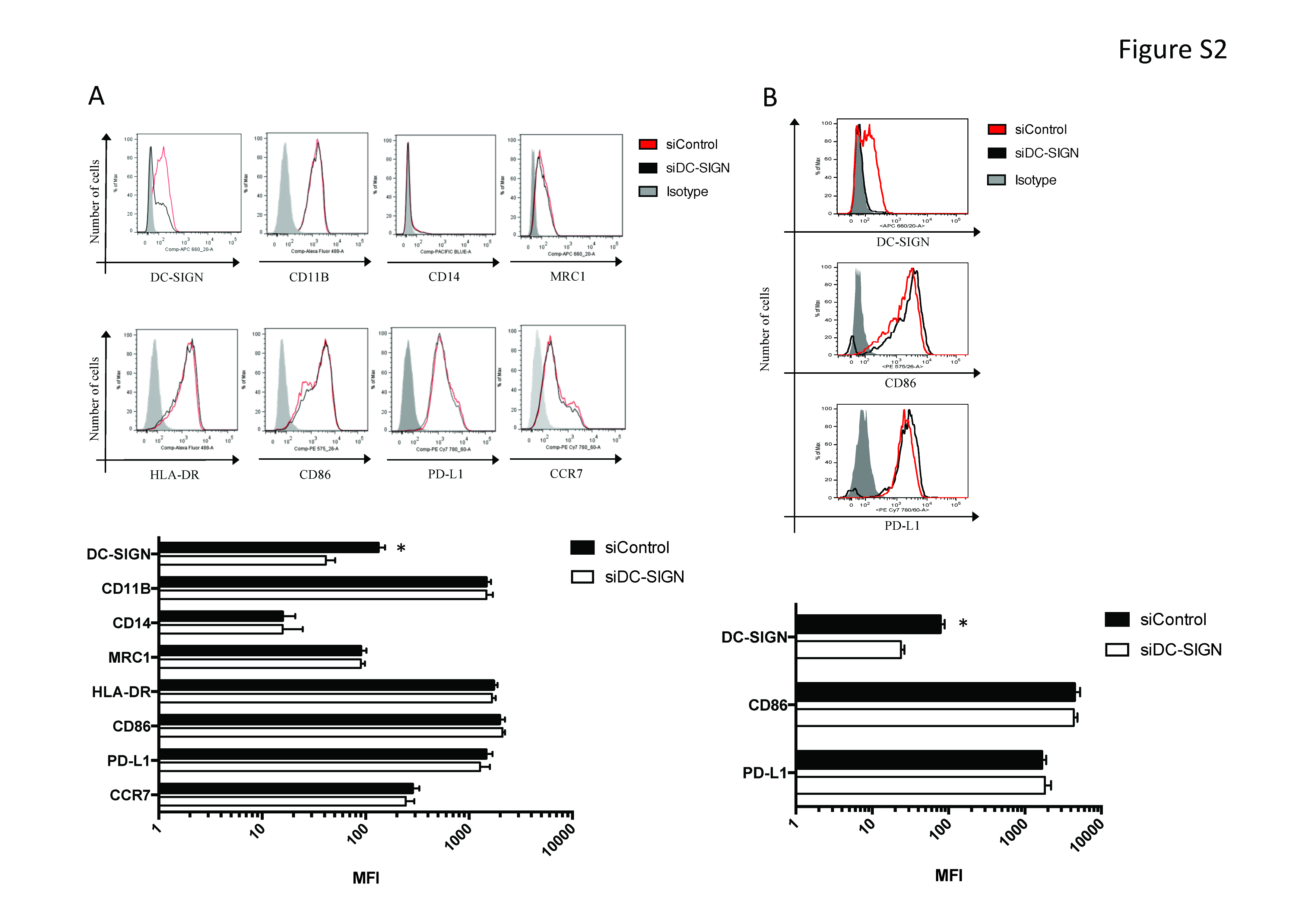

Supplement: Supplementary file 2 [file Image_2.tif]

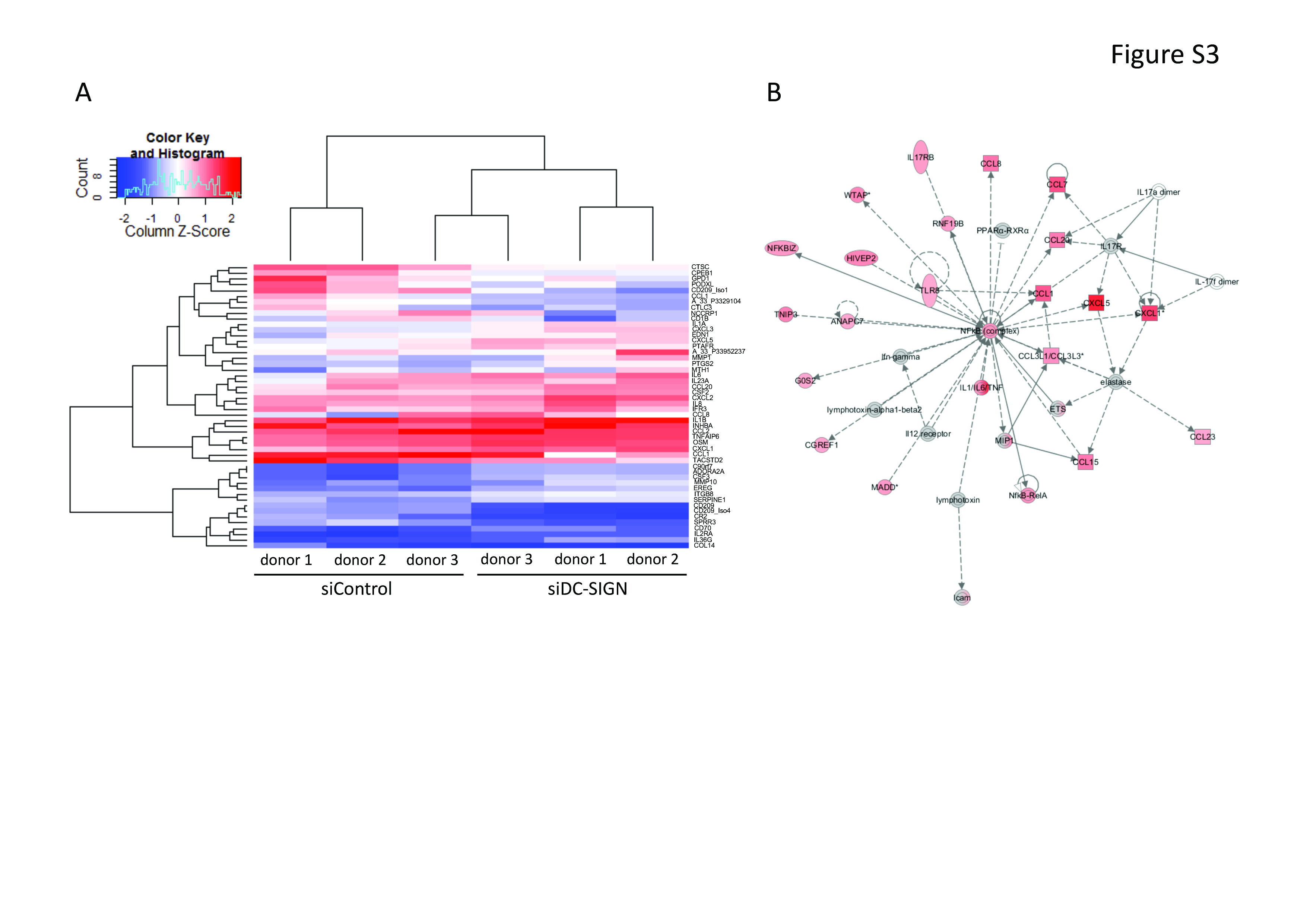

Supplement: Supplementary file 3 [file Image_3.tif]

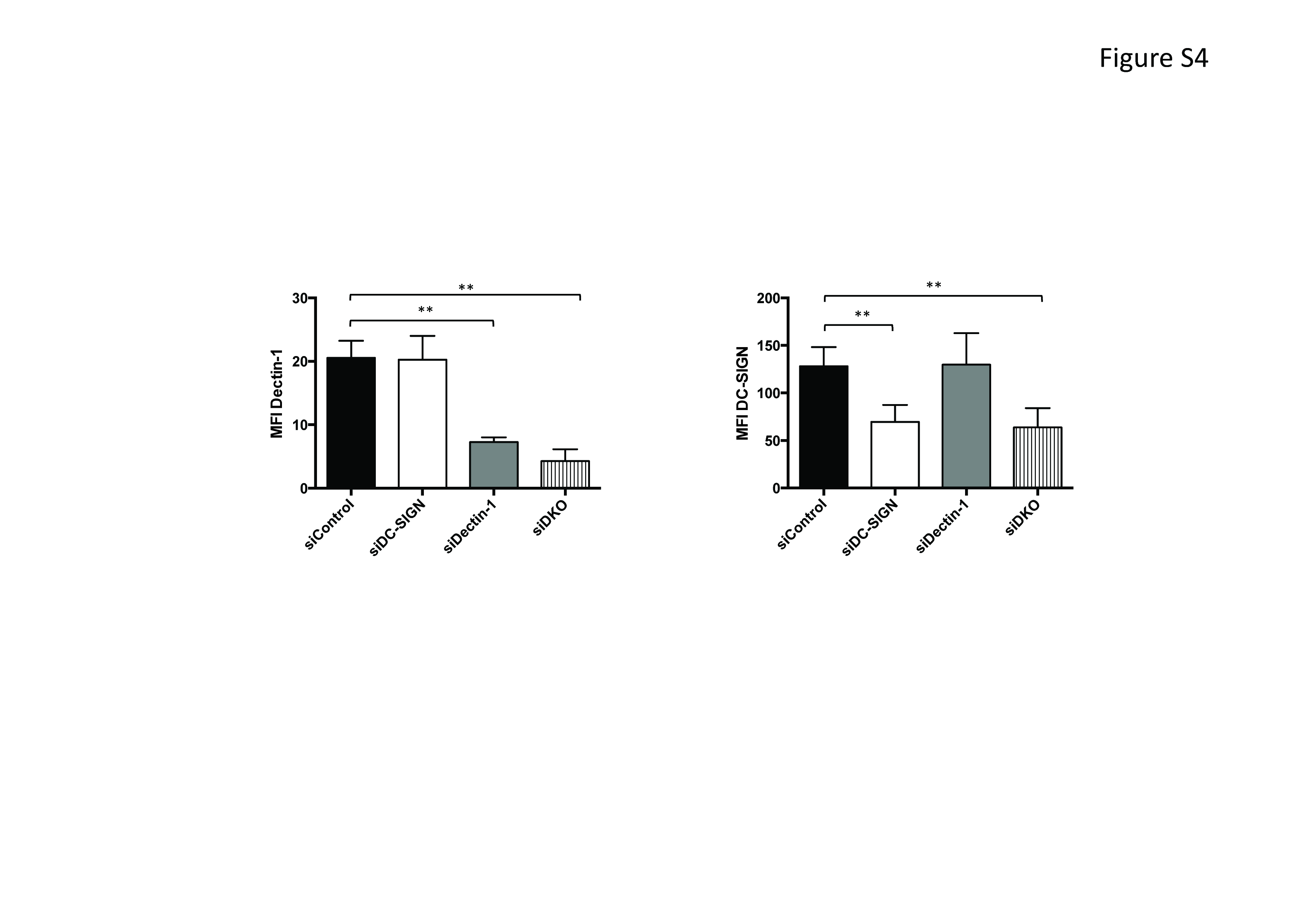

Supplement: Supplementary file 4 [file Image_4.tif]

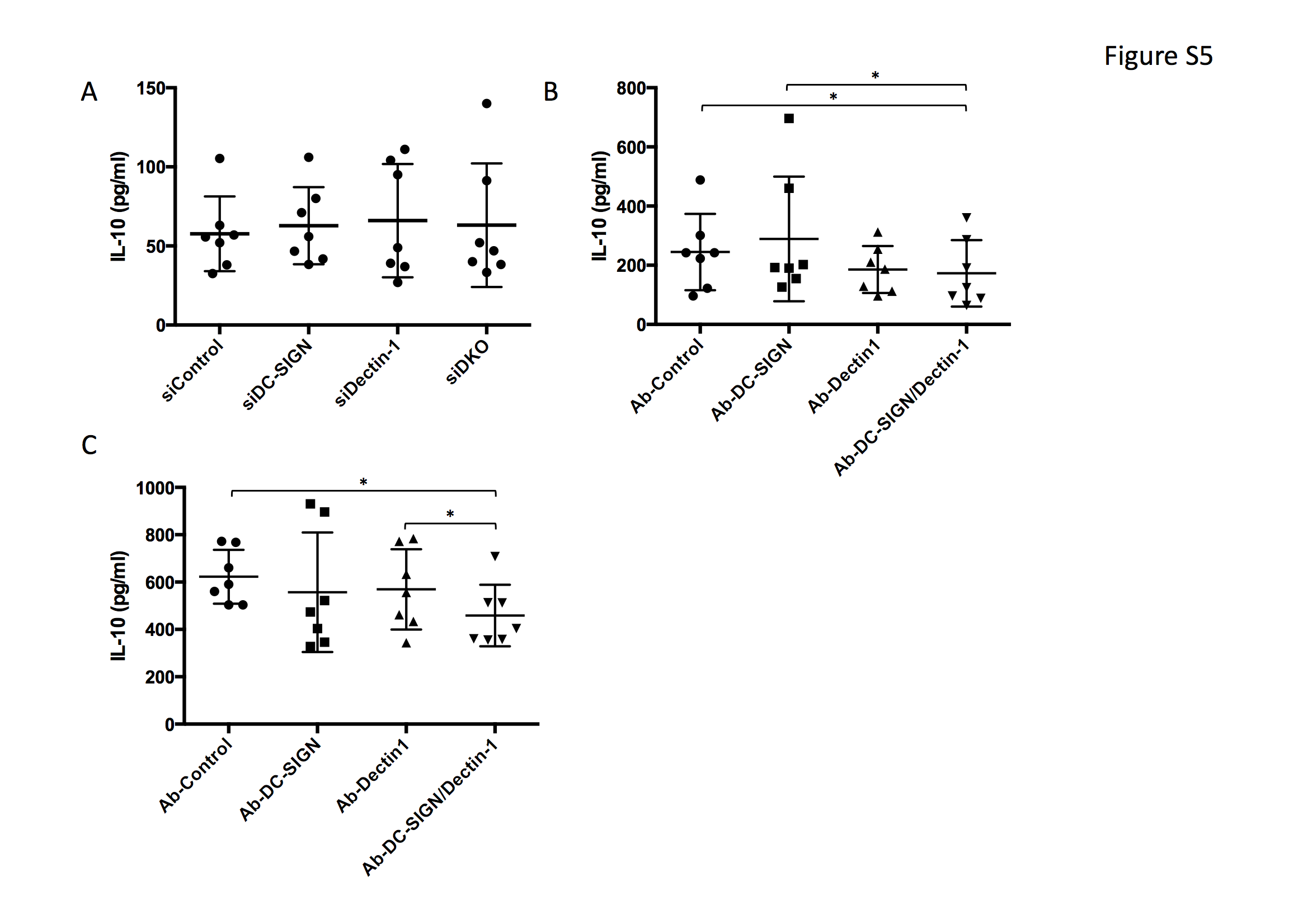

Supplement: Supplementary file 5 [file Image_5.tiff]
