# Supplementary material for: The C-Type Lectin Receptor DC-SIGN Has an Anti-Inflammatory Role in Human M(IL-4) Macrophages in Response to Mycobacterium tuberculosis
Source: Front Immunol. 2018 Jun 12;9:1123. doi: 10.3389/fimmu.2018.01123 (PMC6006465; doi:10.3389/fimmu.2018.01123)
Supplement: Supplementary file 7 [file Presentation_1.PDF]

Supplementary Material

The C-type lectin receptor DC-SIGN has anti-inflammatory role in human M(IL-4) macrophages in response to *Mycobacterium tuberculosis*

Geanncarlo Lugo-Villarino[1,2,3*†], Anthony Troegeler[1,2,3†], Luciana Balboa[2,3,4], Claire Lastrucci[1,2,3,5], Carine Duval[1], Ingrid Mercier[1], Alan Bénard[1,6], Florence Capilla[5], Talal Al Saati[5], Renaud Poincloux[1], Ivanela Kondova[7], Frank A. W. Verreck[7], Céline Cougoule[1,2,3], Isabelle Maridonneau-Parini[1,2,3], Maria del Carmen Sasiain[2,3,4] and Olivier Neyrolles[1,2,3]

[1] Institut de Pharmacologie et de Biologie Structurale, IPBS, Université de Toulouse, CNRS, UPS, Toulouse, France,
[2] International Associated Laboratory (LIA) CNRS "IM–TB/HIV" (1167), Toulouse, France,
[3] International Associated Laboratory (LIA) CNRS "IM–TB/HIV" (1167), Buenos Aires, Argentina,
[4] IMEX-CONICET, Academia Nacional de Medicina, Buenos Aires, Argentina,
[5] INSERM/UPS/US006 CREFRE, CHU Purpan, Toulouse, France,
[6] Department of Surgery, University Hospital Erlangen, Friedrich-Alexander University Erlangen-Nürnberg, Germany.
[7] Biomedical Primate Research Centre, Rijswijk, Netherlands

[†]These authors contributed equally to this work

[*]**Correspondance:** Geanncarlo Lugo-Villarino (lugo@ipbs.fr)

1. Supplementary Tables

Supplementary Table S1: Transcriptome analysis performed in DC-SIGN-depleted and control M(IL-4) macrophages infected with *M. tuberculosis* (see accompanying excel file).

Supplementary Table S2. Top 25 genes that are differentially expressed between DC-SIGN -deficient and control M(IL-4) macrophages at 4 h post-infection with Mtb.

No.	Gene Name	Sytematic Name	Gene Symbol	Ratio (KO*/WT**) M(IL-4)	t-test
1	alpha-inducible protein 27	NM_005532	IFI27	8.78	0.018
2	interleukin 6	NM_005532	IL6	3.38	0.013
3	oncostatin M	NM_020530	OSM	2.91	0.081
4	pentraxin 3	NM_002852	PTX3	2.88	0.012
5	progestin and adipoQ receptor family member V	NM_001104554	PAQR5	2.79	0.032
6	chemokine (C-X-C motif) ligand 1	NM_001511	CXCL1	2.79	0.001
7	interleukin 17 receptor B	NM_018725	IL17RB	2.76	0.038
8	chemokine-like receptor 1	NM_004072	CMKLR1	2.68	0.036
9	legumain	NM_001008530	LGMN	2.60	0.045
10	Fc fragment of IgG, low affinity IIIa, receptor	NM_000569	FCGR3A	2.52	0.001
11	arrestin domain containing 3	NM_020801	ARRDC3	2.48	0.002
12	Fc fragment of IgG, high affinity Ib, receptor	NM_001017986	FCGR1B	2.47	0.001
13	EPH receptor B2	NM_004442	EPHB2	2.46	0.007
14	potassium inwardly-rectifying channel, subfamily J, member 2	NM_000891	KCNJ2	2.43	0.035
15	G protein-coupled receptor 84	NM_020370	GPR84	2.37	0.017
16	selenoprotein P, plasma, 1	NM_005410	SEPP1	2.36	0.034
17	leukocyte immunoglobulin-like receptor, subfamily B (with TM and ITIM domains), member 5	NM_006840	LILRB5	2.33	0.018
18	interleukin 1, beta	NM_000576	IL-1B	2.16	0.043
19	nucleotide-binding oligomerization domain containing 2	NM_022162	NOD2	2.14	0.028
20	LY6/PLAUR domain containing 1	NM_144586	LYPD1	2.11	0.001
21	methyltransferase like 7B	NM_152637	METTL7B	0.49	0.025
22	Fc fragment of IgE, high affinity I, receptor for; alpha polypeptide	NM_002001	FCER1A	0.43	0.036
23	CD1b receptor	NM_001764	CD1B	0.29	0.011
24	chemokine (C-C motif) ligand 17	NM_002987	CCL17	0.27	0.097
25	DC-SIGN receptor	NM_021155	CD209	0.14	0.009

*KO, DC-SIGN-deficient M(IL-4) macrophages; **WT, control M(IL-4) macrophages. This table list a set of genes that are derived from the transcriptomic array analysis. Please consult Table S1 for the full list of DEG.

Table S3. Top 25 genes that are differentially expressed between DC-SIGN -deficient and control M(IL-4) macrophages at 18 h post-infection with Mtb.

No.	Gene Name	Sytematic Name	Gene Symbol	Ratio (KO*/WT**) M(IL-4)	t-test
1	interleukin 6	NM_005532	IL6	7.70	0.081
2	chemokine (C-X-C motif) ligand 3	NM_002090	CXCL3	7.05	0.051
3	chemokine (C-X-C motif) ligand 5	NM_002994	CXCL5	6.65	0.023
4	prostaglandin-endoperoxide synthase 2	NM_000963	PTGS2	6.31	0.053
5	chemokine (C-X-C motif) ligand 2	NM_002089	CXCL2	5.47	0.055
6	chemokine (C-X-C motif) ligand 1	NM_001511	CXCL1	2.79	0.001
7	interleukin 1, alpha	NM_000575	IL1A	4.96	0.023
8	chemokine (C-X-C motif) ligand 1	NM_001511	CXCL1	4.65	0.049
9	inhibin, beta A	NM_002192	INHBA	4.33	0.017
10	chemokine (C-C motif) ligand 7	NM_006273	CCL7	4.02	0.058
11	oncostatin M	NM_020530	OSM	3.97	0.022
12	chemokine (C-C motif) ligand 2	NM_002982	CCL2	3.94	0.063
13	integrin, beta 8	NM_002214	ITGB8	3.92	0.041
14	interferon, alpha-inducible protein 27	NM_005532	IFI27	3.86	0.020
15	tumor necrosis factor, alpha-induced protein 6	NM_007115	TNFAIP6	3.76	0.038
16	chemokine (C-C motif) ligand 1	NM_002981	CCL1	3.75	0.008
17	adenosine A2a receptor	NM_000675	ADORA2A	3.75	0.030
18	phosphodiesterase 4B, cAMP-specific	NM_001037341	PDE4B	3.72	0.034
19	platelet-activating factor receptor	NM_000952	PTAFR	3.70	0.008
20	interleukin 8	NM_000584	IL8	3.66	0.089
21	non-specific cytotoxic cell receptor protein 1 homolog	NM_001001414	NCCRP1	0.474	0.042
22	chloride intracellular channel 3	NM_004669	CLIC3	0.459	0.086
23	CD1b receptor	NM_001764	CD1B	0.421	0.013
24	podocalyxin-like	NM_001018111	PODXL	0.338	0.075
25	DC-SIGN receptor	NM_021155	CD209	0.103	0.043

*KO, DC-SIGN-deficient M(IL-4) macrophages; **WT, control M(IL-4) macrophages. This table list a set of genes that are derived from the transcriptomic array analysis. Please consult Table S1 for the full list of DEG.

Supplementary Table S4: Human primer sequences used in qPCR analysis

Gene name	Forward	Reverse
IL10	5'-ACTTTAAGGGTTACCTGGGTTGC-3'	5'-TCACATGCGCCTTGATGTCTG-3'
DC-SIGN	5'-AAATCAGGAAGGCACGTGGCAAT-3'	5'-TGTTGGGCTCTCCTCTGTTCCAA-3'
TNF	5'-ATGAGCACTGAAAGCATGATCC-3'	5'-GAGGGCTGATTAGAGAGAGGTC-3'
CCL1	5'-CTCATTTGCGGAGCAAGAGAT-3'	5'-GGAGCTGGTATTTCTGTAACACA-3'
FCAR	5'-TGCTACGGTTGGTACAACAGG-3'	5'-AGTTCTGCGTCGTGTAATCTTG-3'
IRAK2	5'-ATGGCCTGCTACATCTACCAG-3'	5'-TAGGAGGCGAACTCCATCCA-3'
SERPINE1	5’-ATTCAAGCAGCTATGGGATTCAA-3’	5’-CTGGACGAAGATCGCGTCTG-3’
IER3	5’-CGGAGCCCTCGGACTACGCT-3’	5’- GGGATACGCTCTCGCGCACC-3’
CCL4	5’-CGCCTGCTGCTTTTCTTACAC-3’	5’-CAGACTTGCTTGCTTCTTTTGG-3’
IL23A	5’-GGACAACAGTCAGTTCTGCTT-3’	5’-CACAGGGCTATCAGGGAGC-3’
IL1b	5’-TTTGAGTCTGCCCAGTTCCC-3’	5’-TCAGTTATATCCTGGCCGCC-3’
OSM	5’-AGACTGGCCGACTTAGAGCA-3’	5’-CCCAGACCTCTCCAAATCCT-3’
CXCL1	5’-AGGGAATTCACCCCAAGAAC-3’	5’- ACTATGGGGGATGCAGGATT-3’
IL-6	5’-CTCGACGGCATCTCAGCC-3’	5’-GCAAGTCTCCTCATTGAATCCAG-3’

2. Supplementary Figure Legends

Supplementary Figure 1. DC-SIGN expression and inactivation in human M(IL-4) macrophages (related to Figure 3-5). **(A)** DC-SIGN is a cell-surface marker of M(IL-4) macrophages. Human monocytes were differentiated into macrophages using M-CSF. At day 5, the cells were activated for 48 h using different stimuli to yield M(LPS+IFNγ, white) and M(IL-4, black) macrophages. At day 7, the cells were harvested and assessed for the indicated cell-surface marker by flow cytometry. Horizontal bar graphs illustrating the median fluorescent intensity (MFI) for the indicated cell-surface markers. Results are expressed as mean $\pm$ SD (n = 6 donors). Statistical significance is provided for the expression of each marker between the cell populations. **(B)** siRNA-mediated gene inactivation of DC-SIGN in M(IL-4) macrophages does not affect cell viability. At day 5, macrophages were transfected with siRNA targeting DC-SIGN (siDC-SIGN, white) or a non-targeting siRNA (siControl, black). The following day, the cells were activated with IL-4 to induce the M(IL-4) program. The cells were then harvested 72 h after transfection, and the expression of DC-SIGN was assessed in non-permeabilized cells by flow cytometry. Left, vertical scatter plots showing the MFI for DC-SIGN expression in siControl and siDC-SIGN macrophages. Results are expressed as mean $\pm$ SD (n = 6 donors); each circle within the vertical scatter plots represents a single donor. Right, vertical bar graphs depict the percentage of live cells positive in siControl and siDC-SIGN 72 h post-transfection, as determined by using Annexin-V-FITC kit designed for flow cytometry. Results are expressed as mean $\pm$ SD (n = 6 donors). Two-tailed Wilcoxon (matched-paired/nonparametric): $* P < 0.05$; *NS,* not significant.

Supplementary Figure 2. Depletion of DC-SIGN expression does not affect the establishment of the M(IL-4) macrophage program (related to Figure 3-5, and Figure S1). Human monocytes were differentiated into macrophages using M-CSF. At day 5, macrophages were transfected with siRNA targeting DC-SIGN (siDC-SIGN) or a non-targeting siRNA (siControl). The following day, the cells were activated with IL-4 to induce the M(IL-4) program. **(A)** Normal establishment of the M(IL-4) program. The cells were then harvested 72 h after transfection, and the cell-surface expression of the indicated markers was assessed in non-permeabilized cells by flow cytometry. Top, histograms showing the MFI for the indicated marker in siDC-SIGN (red) and siControl (black) M(IL-4) macrophages; the background staining is shown based on the isotype control (grey) for each given marker. Representative of 4 independent experiments. Bottom, horizontal bar graphs illustrating the MFI for the indicated cell-surface markers in siDC-SIGN (white) and siControl (black) M(IL-4) macrophages. Results are expressed as mean $\pm$ SD (n = 4 donors). **(B)** Normal upregulation of co-stimulated markers in M(IL-4) macrophages in response to LPS. After activation with IL-4, macrophages were then challenged with LPS for 24 h, and the cell-surface marker expression was assessed by flow cytometry. Top, histograms showing the MFI for the indicated marker in siDC-SIGN (red) and control (black) M(IL-4) macrophages; the background staining is shown based on the isotype control (grey) for each given marker. Representative of 4 independent experiments. Bottom, horizontal bar graphs illustrate the MFI for the indicated cell-surface markers in siDC-SIGN (white) and siControl (black) M(IL-4) macrophages. Results are expressed as mean $\pm$ SD (n = 4 donors). Two-tailed Wilcoxon (matched-paired/nonparametric): $* P < 0.05$.

Supplementary Figure 3. DC-SIGN expression in M(IL-4) macrophages modulates the pro-inflammatory response against Mtb (related to Figure 3). **(A)** Heatmap of some of the most differentially expressed genes (DEG) in control (siControl) versus DC-SIGN-depleted (si-DCSIGN) M(IL-4) macrophages after Mtb infection for 18 h. For each gene, the relative gene expression value is depicted across from control (left columns) to DC-SIGN-depleted (right columns) macrophages, and ranging from low (blue) to high (red). DEG was determined based on a false discovery rate (*t*-test, $p < 0.1$) and threshold of a two-fold change in the comparison between the two conditions. Each column represents one independent experiment derived from a single donor. **(B)** DEG analysis with Ingenuity Pathway software. NFκβ signaling as an example of the main networks revealed by Ingenuity pathway analysis depicting the interconnections of some of the most significant upregulated genes (red) in DC-SIGN-depleted M(IL-4) macrophages, as compared to control cells.

Supplementary Figure 4. Simultaneous siRNA-mediated gene inactivation of DC-SIGN and Dectin-1 in M(IL-4) macrophages (related to Figure 5). **(A)** Macrophages differentiated at day 5 were transfected with siRNA targeting DC-SIGN (siDC-SIGN, white) and Dectin-1 (siDectin-1, grey), both siRNAs (siDKO, vertical stripes), or a non-targeting siRNA (siControl, black). The following day, the cells were activated with IL-4 to induce M(IL-4) program and the CLR expression. After 72 h, the cells were harvested and the expression for Dectin-1 (left) and DC-SIGN (right) was assessed by flow cytometry. The vertical bar graphs illustrate the MFI of DC-SIGN and Dectin-1 for each condition. Results are expressed as mean as mean $\pm$ SD ($n = 6$ donors). Two-tailed Wilcoxon (matched-paired/nonparametric): * $P < 0.05$, ** $P < 0.01$.

Supplementary Figure 5. Assessment of IL-10 production during the crosstalk between DC-SIGN and Dectin-1 (related to Figure 5). (A) Upon siRNA-mediated gene silencing of either DC-SIGN (siDC-SIGN) or Dectin-1 (siDectin-1), or both (siDKO), M(IL-4) macrophages infected with Mtb at MOI of 3 bacteria to 1 cell. At 18 h p.i., the supernatant from these cells was collected, and the production of IL-10 was measured by ELISA analysis. Results are expressed as vertical scatter plots, and as mean + SD (n = 7 donors). **(B-C)** Prior to stimulation, M(IL-4) macrophages were pre-treated for 30 min with blocking antibodies for either DC-SIGN or Dectin-1, or both. An irrelevant antibody was used as a control. M(IL-4) macrophages were then treated with either **(B)** cytochalasin D (1 μg/ml), β-glucan (10 μg /ml) and ManLAM (10 μg/ml), or **(C)** LPS (1 μg/ml) and ManLAM (10 μg/ml). After 24 h, the supernatants were collected and the production of IL-10 was measured by for ELISA analysis.
